# Evaluating the Evolvability of *Paranthropus* Cranial Morphology in Relation to Feeding Biomechanics

**DOI:** 10.1002/ajpa.70136

**Published:** 2025-10-20

**Authors:** Hyunwoo Jung, Campbell Rolian, David S. Strait, Karen L. Baab

**Affiliations:** ^1^ Department of Anatomy College of Graduate Studies, Midwestern University Glendale Arizona USA; ^2^ Department of Anthropology University of California, One Shields Avenue Davis California USA; ^3^ Department of Anatomy and Cell Biology McGill University Montreal Quebec Canada; ^4^ Department of Anthropology Washington University in St. Louis St. Louis Missouri USA; ^5^ Palaeo‐Research Institute, University of Johannesburg Johannesburg South Africa; ^6^ DFG Center for Advanced Studies “Words, Bones, Genes, Tools,” University of Tübingen Tübingen Germany

**Keywords:** biomechanical specializations, evolutionary quantitative genetics, hominins, morphological evolution, viability selection simulation

## Abstract

**Objective:**

Although disagreement persists as to the precise nature of the diet of *Paranthropus*, there is a consensus that the food resources consumed by *Paranthropus* were in some way mechanically challenging to process (i.e., by being “hard” and/or “tough”). While the highly derived feeding apparatus of *Paranthropus* likely conferred biomechanical performance advantages while consuming certain types of foods, it may also have limited the ability of these early hominins to respond to selection and evolve rapidly toward new adaptive peaks (i.e., reduced their evolvability).

**Materials and Methods:**

We employed viability selection modeling to test this hypothesis. Viability selection simulations were performed using *Paranthropus boisei* (OH 5), *Australopithecus afarensis* (A.L. 444‐2), and *Homo habilis* (KNM‐ER 1813) specimens. We simulated the generation‐to‐generation evolution of biomechanically informative linear dimensions in a population where an individual's probability of survival (i.e., viability) was determined by its distance to a predetermined adaptive peak. The number of generations required for an evolving population to reach a new adaptive peak was used as a measure of evolvability.

**Results:**

The results showed that the mean number of generations from *P. boisei* to *H. habilis* was larger than in the reverse direction when modeled using either chimpanzee or human estimates of population variance/covariance. It took longer for *P. boisei* to evolve toward *Au. afarensis* than in the reverse direction, but only with the chimpanzee estimates of population variance/covariance.

**Discussion:**

The results suggest that *P. boisei* faced limitations in cranial evolvability, particularly if selection favored a cranial morphology similar to *H. habilis* .

**Summary**

- Highly derived cranial features in *Paranthropus boisei* may have constrained their macroevolutionary evolvability.
- Evolution of trait variance/covariance matrices can result in either a concordance or a discrepancy between micro‐ and macroevolutionary evolvability.

## 1 Introduction

Two major hominin clades (*Homo* and *Paranthropus*) emerged and diversified between 3 and 2 million years ago, likely in response to global cooling and the development of increasingly open, arid, and/or seasonal environments (Vrba 1985, 1995; Harrison 2011; Potts 2012; Wood and Boyle 2016; Robinson et al. 2017; Herries et al. 2020; Martin et al. 2021). Species in the genus *Paranthropus* (known informally as robust australopiths) garner research interest as their distinctive skull morphology provides a valuable opportunity to unravel the relationship between environmental change and feeding/dietary adaptations in hominins (and mammals more broadly) (e.g., Jolly 1970; Walker 1981; Peters 1987; Teaford and Ungar 2000; Wood and Strait 2004; Scott et al. 2005; Wood and Constantino 2007; Ungar et al. 2008; Cerling et al. 2011; Daegling et al. 2013; Strait et al. 2013; Smith et al. 2015; Martin et al. 2021).

*Paranthropus* specimens exhibit distinctive skull features, most exaggerated in *P. boisei*, including a flaring and anteriorly positioned zygoma, a sagittal crest, large infratemporal fossa, a robust mandibular corpus/symphysis, a tall mandibular ramus, massive postcanine tooth size (i.e., megadontia), and exceptionally thick tooth enamel (e.g., Broom 1938; Robinson 1954; Tobias 1967; Rak 1983; McHenry 1984; Kimbel et al. 1984; Walker et al. 1986; Grine 1988; Grine and Martin 1988; Grine and Daegling 1993; Keyser 2000; Kimbel et al. 2004; Wood and Constantino 2007; Olejniczak et al. 2008; Skinner et al. 2015; Rak et al. 2021; Martin et al. 2021). These features have been suggested as evidence for increases in the mechanical advantage of the jaw lever system, large jaw adductor muscles, resistance to high stresses generated during feeding, and tooth crowns that are resistant to wear and/or fracture (e.g., Rak 1983; Demes and Creel 1988; Hylander 1988; Teaford and Ungar 2000; Lucas 2004; Daegling et al. 2013; Strait et al. 2013; Smith et al. 2015; Ungar and Hlusko 2016; Sponheimer et al. 2023). Indeed, a finite element analysis (FEA) study reported that the *P. boisei* cranium (OH 5) exhibited lower strains at many cranial locations during molar and premolar biting compared to *Australopithecus africanus* (Sts 5) and *Pan troglodytes* , even when muscle and bite forces were twice as high or greater in *P. boisei* (Smith et al. 2015). Consequently, the “hyper‐robust” skull morphology found in *Paranthropus* could have functioned to effectively comminute food items that are “hard” (i.e., stiff and crack resistant) and/or “tough” (i.e., compliant and crack resistant) based on its capability to generate and withstand high magnitude and/or repetitive loads (Hylander 1988; Lucas 2004; Wood and Constantino 2007; Daegling et al. 2013; Strait et al. 2013; Smith et al. 2015; Ungar and Hlusko 2016).

### 1.1 Potential Evolutionary Consequences of *Paranthropus* Cranial Morphology

Despite ongoing debates about diet (e.g., Daegling et al. 2013 vs. Strait et al. 2013), there is no doubt that *Paranthropus* was morphologically derived (see Supplementary S1 for further discussion). It is possible that certain trait combinations emerged during the evolution of *Paranthropus* that were, in turn, difficult to rapidly decouple and modify in response to new selective pressures, thus “trapping” these species in a region of morphospace that slows the tempo of evolutionary change away from this adaptive peak (Villmoare 2013; Rolian 2020). In other words, highly derived, integrated traits may have led to a loss of evolvability in robust australopiths.

In the present study, we investigated whether the highly derived cranial morphology of *P. boisei* might have resulted in a disadvantage for morphological evolution toward new adaptive peaks. To address this issue, we focused on the evolvability of cranial dimensions related to feeding biomechanics in *Paranthropus*. Here, the evolvability of traits refers to the capability or potential to evolve in response to selection (reviewed in Hansen et al. (2023)). Measures of evolvability often quantify genetic (or phenotypic) variation in traits relative to their pattern and magnitude of (co)variation (e.g., Hansen and Houle 2008) or genetic/mutational variability (e.g., Wagner and Altenberg 1996). However, challenges arise due to the limited sample size for reliable estimation of genetic/phenotypic variation and the absence of a mutation matrix for biomechanically informative measurements in the fossil hominins used in this study.

We employed in silico simulations, specifically viability selection modeling, within an evolutionary quantitative genetics framework (Bürger and Lynch 1995; Jones et al. 2003, 2004, 2007; Salazar‐Ciudad and Marín‐Riera 2013; Rolian 2020, 2024). Viability selection modeling simulates the evolution of a taxon toward a new adaptive peak by eliminating those individuals from the evolving population each generation who are among those farthest from the new peak. For instance, Rolian (2020) assessed the evolvability of primate limb bone morphology in terms of the number of generations required to reach alternative adaptive peaks. This work showed that the more derived limb morphology in hominoids relative to cercopithecoids may reduce their evolvability, thereby increasing the probability of extinction.

More familiar measures of (conditional) evolvability and autonomy/integration, as proposed by Hansen and Houle (2008), are indicative of microevolutionary evolvability and constraint, whereas viability selection modeling provides a way to investigate evolutionary potential over macroevolutionary timescales (Rolian 2020). Specifically, microevolutionary evolvability reflects a phenotype's capacity to evolve in any (or a specific) direction within morphospace in response to selection over single generations (Hansen and Houle 2008). In the context of viability selection modeling, macroevolutionary evolvability represents the evolutionary rate toward new adaptive peaks in morphospace under repeated generation‐to‐generation viability selection, rather than just the magnitude or direction of the response of a single episode of selection (Rolian 2020).

Empirical studies in primates have demonstrated that patterns of trait variance and covariance may influence not only microevolution but also macroevolutionary dynamics (see von Cramon‐Taubadel (2022) and Auerbach et al. (2023) for a review). This includes taxonomic divergence in craniodental morphology within primate clades, such as primate teeth (Mongle et al. 2022; Machado et al. 2023), platyrrhine crania (Marroig and Cheverud 2005), cercopithecoid crania (Schroeder et al. 2022), hominoid crania (Schroeder and von Cramon‐Taubadel 2017), and hominin crania (Ackermann 2002; Roseman et al. 2011; Baab 2018). Assessing the role of population‐level trait variance and covariance in macroevolution (i.e., transition between micro‐ and macroevolutionary scales) generally relies on the assumption that variance/covariance (V/CV) structures remain consistent along phylogenetic branches connecting both ancestral and descendant taxa, even over timescales of millions of years (Schluter 1996; Arnold et al. 2001; Ackermann 2002; Marroig and Cheverud 2005; Marroig et al. 2009; Roseman et al. 2011; Schroeder and von Cramon‐Taubadel 2017; Baab 2018; Schroeder et al. 2022). For instance, from a phylogenetic comparative perspective, this assumption has been supported by findings that mammalian crania, including primates, exhibit similar V/CV structures (e.g., patterns of integration) despite considerable phylogenetic and morphological distances (e.g., Marroig et al. 2009; Porto et al. 2009). However, it may overlook potential fluctuations or shifts in trait V/CV structures deep in evolutionary time along phylogenetic branches, even when similar patterns are observed at the tips of the tree. Viability selection modeling provides a methodological framework for addressing this issue by allowing the evolution of trait V/CV structures during macroevolutionary shifts toward new adaptive peaks, which may be biologically more realistic (Bürger and Lynch 1995; Jones et al. 2003, 2004, 2007; Salazar‐Ciudad and Marín‐Riera 2013; Rolian 2020, 2024).

In this study, following a similar modeling approach to Rolian (2020, 2024), we tested the hypothesis that the derived cranial morphology associated with feeding biomechanics in *P. boisei* may have substantially constrained their macroevolutionary evolvability compared to *Au. afarensis* and *H. habilis*. Furthermore, the study examined how (micro)evolutionary parameters in population V/CV structures are associated with macroevolutionary evolvability, defined as the number of generations required to reach a new adaptive peak (i.e., a proxy for the evolutionary rate).

## 2 Materials and Methods

### 2.1 Skeletal and Fossil Samples

Skeletal samples of extant taxa included adult specimens of *H. sapiens* , *Pan troglodytes troglodytes* , and *P. t. schweinfurthii* (Table 1). We determined the adult status based on the presence of fully erupted third molars and/or the (complete) fusion of the spheno‐occipital synchondrosis. Three‐dimensional scan data of the skeletal samples were acquired using various scanning technologies, including a Creaform ExaScan 3D laser scanner (Creaform, Québec, Canada), a Breuckmann optical white light scanner (Breuckmann GmbH, Braunschweig/Meersburg, Germany), an HDI‐120 structured‐light scanner (LMI Technologies INC, Vancouver, Canada), and an Artec Space Spider scanner (Artec 3D, Senningerberg, Luxembourg). *Homo sapiens* specimens are from an archaeological cemetery from the Meroitic period (c. 350 bce to ad 350) Nubian site of Semna South, located along the upper Nile River in Sudan. These specimens are housed in the School of Human Evolution and Social Change at Arizona State University. Most of the *P. t. troglodytes* and *P. t. schweinfurthii* crania are from the Cleveland Museum of Natural History (OH, USA) and the Royal Museum for Central Africa (Tervuren, Belgium), as well as the American Museum of Natural History (NY, USA), the Field Museum of Natural History (IL, USA), the Museum of Comparative Zoology (MA, USA), and the Smithsonian Museum of Natural History (Washington, D.C., USA). Most chimpanzee surface scan data were provided by Jason Massey (Monash University) and Kieran McNulty (University of Minnesota). Some human surface scan data were contributed by Claire Terhune (University of Arkansas). The remainder of the Semna South human sample was scanned by the authors for the current project, while the rest of the chimpanzee sample was scanned for a previous project (Jung 2021).

**TABLE 1:** Skeletal sample of extant taxa.

Genus	(Sub)species	Female	Male	Total
*Homo*	*sapiens*	52	67	119
*Pan*	*troglodytes troglodytes*	67	26	62
	*troglodytes schweinfurthii*	5	14	19

Three fossil hominins were used as representatives of hypothetical locations in morphospace corresponding to “hypertrophied” (OH 5; *P. boisei*), “ancestral australopith” (A.L. 444‐2; *Au. afarensis*), and “reduced masticatory” (KNM‐ER 1813; *H. habilis* ) taxa. We utilized virtually reconstructed 3D surface models of A.L. 444‐2 (Ledogar et al. 2022), OH 5 (Benazzi et al. 2011), and KNM‐ER 1813 (Benazzi et al. 2014) to maximize the number of linear measurements. The three fossils we studied were among the most complete and had existing 3D reconstructions available that permitted more complete data collection. It is important to note that each fossil specimen was considered representative of its respective species' mean morphology, making each an adaptive peak in each simulation scenario. Obviously, this assumption is unlikely to be true, but we adopt it as a heuristically reasonable simplifying assumption that is a concession to limited sample sizes in most fossil hominin taxa.

### 2.2 Linear Distance Measurements

**FIGURE 1:**
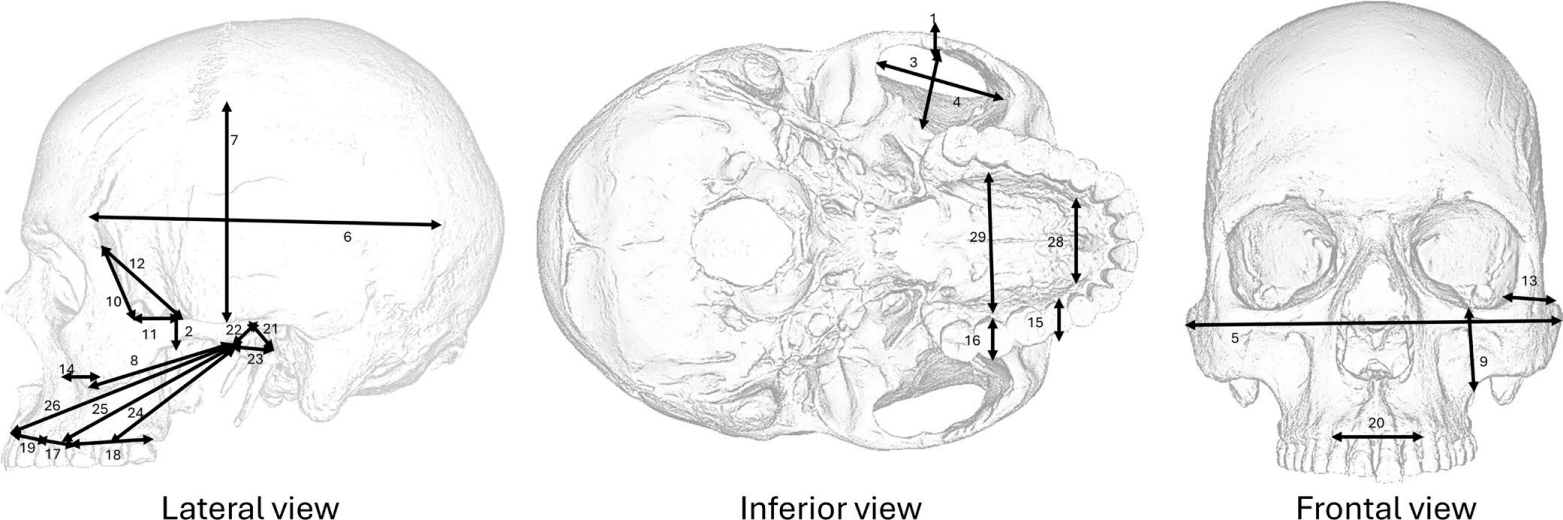
Linear measurements depicted in the human cranium used in this study.

We used 29 linear dimensions in the cranium designed to capture aspects of the triangle of support (defined by the bite point and the working‐ and balancing‐side temporomandibular joints), jaw adductor muscle size, bony reinforcement, and tooth crown mechanics (Figure 1; Tables S1 and S2) (Greaves 1978; Demes and Creel 1988; Hylander 1988, 2013; Spencer 1999; Lucas 2004; Lieberman 2011; Strait et al. 2013; Jung et al. 2023, 2024). Landmarks forming the end points of the linear dimensions were collected from the left side of the cranium or from the right side if it was better preserved, using 3D Slicer v.4.11.20210226. (Fedorov et al. 2012; Table S1). The calculation of linear distances was conducted using the “interlmkdist” function in the geomorph package v.4.0.3. (Baken et al. 2021) in R v.4.1.1. statistical environment (R Core Team 2022). In the case of extant taxa, we adjusted sex‐related variation by subtracting the vector of mean differences × 0.5 from males and adding it to females (see Marroig and Cheverud (2004) and Rolian (2009) for a similar approach). The same approach was applied to account for subspecies variation within *P. troglodytes* . Subsequently, the linear measurements, following the sex (and subspecies) correction procedures, were utilized to generate V/CV or correlation matrices. The intraobserver error, expressed as the mean coefficient of variation, was 1.59%, which falls within acceptable levels (≤ 5%; White and Folkens 2000). Please refer to Jung et al. (2023) for more comprehensive information regarding intra‐observer error and missing landmark estimation in the extant taxa.

### 2.3 Analytic Methods

Viability selection simulations were conducted in MATLAB R2024 (Natick, MA) following Rolian (2020, 2024) with certain modifications required to incorporate fossil data. To conduct the simulation, a random population of 1000 individuals was generated for the evolving (or source) taxon (i.e., the species evolving under viability selection) and target taxon (i.e., the adaptive peak in the simulation) using the measurements of the representative fossil as the target population means and parameterized using the trait phenotypic variance–covariance pattern of the extant taxa (V/CV matrix). The evolution of novel morphology can be associated with changes to the pattern of morphological integration (e.g., upright walking involved changes to pelvis integration; Grabowski et al. 2011), and it is possible that *Paranthropus* had a distinct integration pattern. However, this cannot be evaluated directly as measuring population parameters requires large sample sizes not available in fossil species. Thus, it is necessary to use proxy or estimated V/CV matrices for fossil species. The use of extant hominid V/CV structure as a proxy for fossil hominins is grounded in our previous study, which demonstrated the generally conserved nature of functional morphological integration in the hominine skull using the same measurements as the current study (Jung et al. 2023). The use of close phylogenetic proxies is empirically supported by the generally conserved nature of V/CV structure in mammals (Marroig et al. 2009; Porto et al. 2009), hominoids specifically (Mitteroecker and Bookstein 2008; Singh et al. 2012), and across some *Homo* species (Bookstein et al. 2003; Gunz and Harvati 2007; Roseman et al. 2011). There is also evidence that differences in V/CV matrices or measures of integration/evolvability reflect phylogenetic distance in primates (de Oliveira et al. 2009; Villamil 2021) and specifically within humans and African apes (Ackermann 2002).

Nevertheless, the modeled V/CV matrices in the current study are not static and are both tailored to reflect trait means in the extinct species (see below) and allowed to evolve throughout the simulation. Directly using the extant hominine V/CV structure as a proxy for extinct hominins may be problematic as the amount of variation in some small measurements (e.g., the breadth and height of the midzygomatic arch) in chimpanzees or humans can lead to negative values when generating a normally distributed, multivariate population of *H. habilis* (i.e., 1000 individuals), as the latter has smaller values in those small measurements compared to extant species in this study. Indeed, the simulations could not proceed using the human or chimpanzee V/CV matrix as a proxy for *H. habilis* as the target taxon due to the generation of negative values when simulating the target population of 1000 individuals. This phenomenon primarily reflects a limitation of the function used to derive multivariate normal samples when some of the variables' means are small, but their variances/covariances are not scaled appropriately.

Therefore, it was necessary to adjust the human‐ or chimpanzee‐based V/CV matrices to match expected trait variances in each fossil taxon. First, species‐specific log‐linear regression analyses were conducted using the means and standard deviations of 29 measurements from humans or chimpanzees. The means and standard deviations were natural log‐transformed to establish a linear relationship between them. The regression analysis indicates a strong positive correlation between means and standard deviations across cranial variables (i.e., larger means have larger variances), yielding an adjusted R^2^ value of 0.82 and 0.76 for the human and chimpanzee data, respectively (Figure S1). In the interaction test, the slope (*p* = 0.4) and intercept (*p* = 0.16) were not significantly different between the two regression models. Next, these regression equations were used to predict standard deviations for the 29 measurements in each fossil taxon. The predicted standard deviations were stored in a diagonal matrix (D), which was used to scale the original human or chimpanzee correlation matrix (**R**) as follows: ${\mathrm{VCV}}_{e}=DRD$, where VCV_e_ represents the estimated V/CV matrix of a fossil taxon. Since we used the same human or chimpanzee correlation matrix for each evolutionary scenario, the evolving and target fossil taxa share the same trait correlation patterns, but with magnitudes adjusted for differences in the scale of the measurements in fossil and extant taxa.

#### 2.3.1 Steps of Viability Selection Simulation for Each Generation

The simulation is conducted for each generation through four steps: (1) fitness estimation in the evolving population; (2) culling low‐fitness individuals; (3) adjusting trait (co)variance to prevent depletion of trait (co)variance; and (4) generation of a new evolving population (i.e., in subsequent generations) based on remaining individuals and adjusted trait (co)variance. The simulation completes upon meeting two specific criteria (see below).

After generating the initial evolving and target populations as described above, Mahalanobis distances were calculated from each individual in these two populations to the multivariate mean of the target (fossil) taxon using the estimated V/CV matrix of the target population. The Mahalanobis distances in the evolving population were used as measures of individual fitness, as greater Mahalanobis distance from the target mean increases the likelihood of being culled in the next step. The Mahalanobis distance is a preferable proxy for individual fitness in this simulation compared to purely geometric measures of distance in multivariate space (e.g., Euclidean distances), because it accounts for the phenotypic V/CV structure (e.g., the dispersion of traits in morphospace) of the target population.

For the second step, 20% of individuals were culled from the evolving population of 1000 individuals. Individuals were randomly selected from the cull pool, meaning they had an equal probability of being culled while in the cull pool. This introduces stochasticity into the culling process, reflecting the biological reality that individuals are not eliminated based strictly on their precise distance from the adaptive peak. The pool initially included 95% of individuals farthest from the target mean (i.e., all but the top 5% of individuals closest to the mean morphology could potentially be culled). The pool decreased linearly to 22% of individuals farthest from the target mean over “evolutionary time” to mimic a situation where directional selection operates throughout the simulation, followed by stabilizing selection once the evolving population is close to a new adaptive peak (see fig. S2 in Rolian 2024).

For the third step, trait (co)variance was adjusted to ensure that variance for each trait is not depleted after culling individuals with lower fitness. New trait variances are calculated as follows: $V\left(g\right)=V_{s}+\left(V_{t}-V_{s}\right)\times \left(1-\frac{E\left(g\right)}{E_{o}}\right)$, where V(g) is a given trait's adjusted variance at a given generation (g), V_s_ and V_t_ are the trait's source and target variances, respectively, E(g) is the Mahalanobis distance after the cull event for that generation, and E_o_ is the original Mahalanobis distance between the source and target species means (Rolian 2024, 4). Then, the V/CV matrix of the evolving population was modified using the trait's adjusted variance as calculated above. For this, a correlation matrix of the evolving population after the cull event and diagonal matrix with the square root of the adjusted trait variances (i.e., standard deviations) were used as follows: 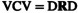, where VCV is the new variance/covariance matrix of evolving population, D is diagonal matrix of traits' adjusted standard deviations, and **R** is the correlation matrix of the evolving population.

In the fourth step, a new source population of 1000 individuals is generated based on the newly calculated multivariate mean and V/CV matrix from Step 3. The target population is also regenerated at each generation, ensuring that it is not static or immutable across generations. Thus, the Mahalanobis distances of individuals in the target population also vary stochastically within a limited range. However, the V/CV structure and measurement means of the target population remain unchanged in the simulation process, which guarantees that the calculation of the Mahalanobis distance between the evolving population and the target morphology is unaffected. The regeneration of the target population at each generation can influence the first criterion for completing the simulation, as it is based on the 95% range of the distribution of Mahalanobis distances between target individuals and their own mean, as described below.

**FIGURE 2:**
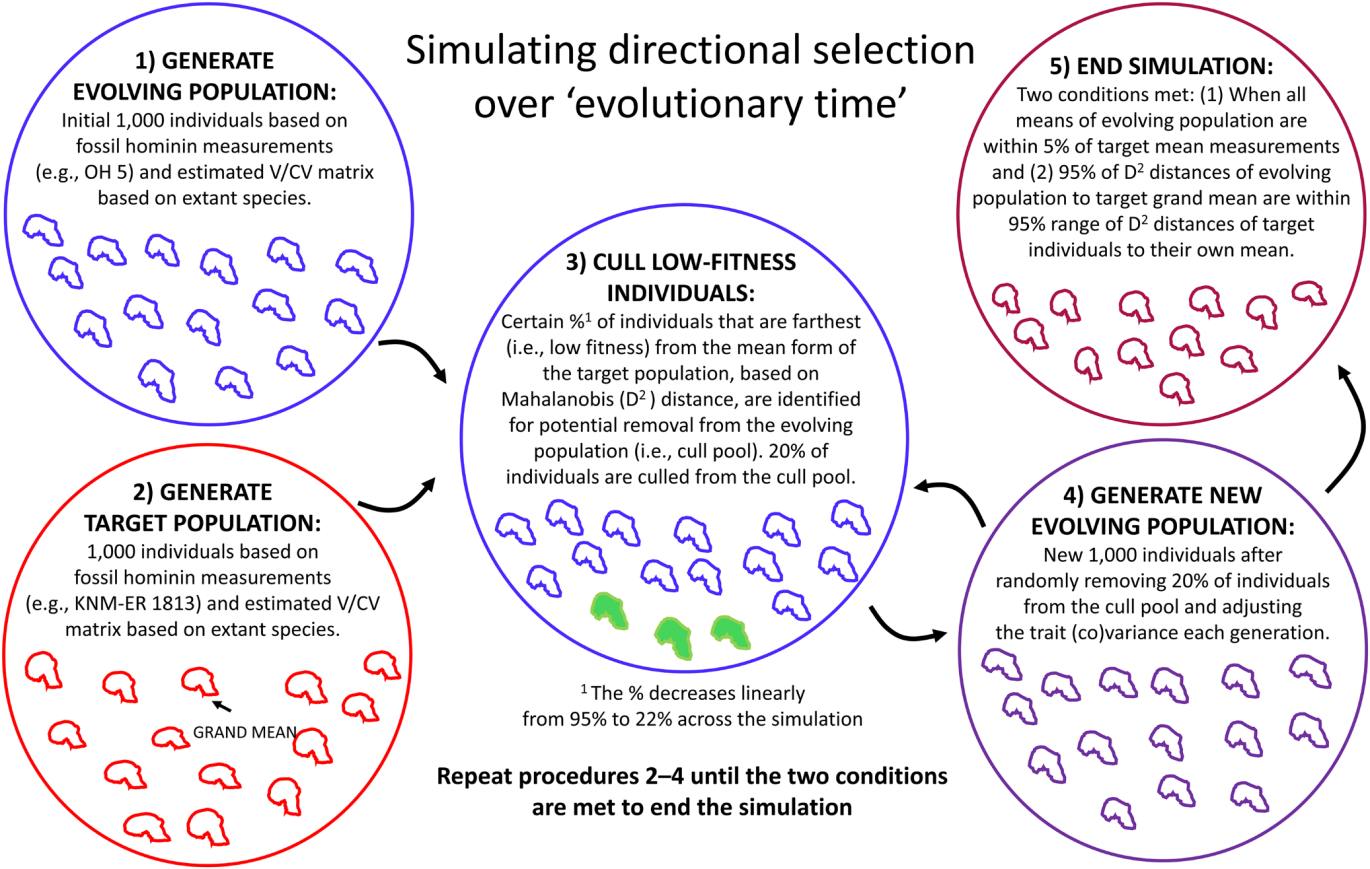
Viability selection simulation procedures. Steps 1–3 of the simulation for each generation, as described in the main text, are combined and represented as Procedure 3 in this schematic figure. In this figure, Procedure 1 involves drawing the initial evolving population, while Procedure 2 involves drawing the target population. Then, the initial evolving population undergoes culling in Procedure 3, and a new evolving population is generated in Procedure 4. If the new population does not meet the two criteria, it returns to Procedure 3. The new evolving population from Procedure 4 and the new target population from Procedure 2 are then used for Procedure 3. At this point, Procedure 1 is no longer required, as we already have a new evolving population from Procedure 4.

This process is repeated each generation of the simulation until two criteria are met: (1) 95% of individual Mahalanobis distances in the source (i.e., evolving) population are within the upper bound of the 95% range of Mahalanobis distances of the target individuals to their own mean (i.e., the mean plus two standard deviations); (2) all trait means under selection in the evolving population are within 5% of the target species' means (Figure 2). In other words, our criteria require the evolving population to closely match both the target species' mean and population distribution (Rolian 2020, 2024).

#### 2.3.2 Duration and Outcome of Simulations

Viability selection simulations were conducted 1000 times per scenario to generate a distribution of simulation outcomes. We recorded the mean number of generations required for the source population to “reach” the target population in these 1000 simulations. Additionally, we presented standardized values calculated as the mean number of generations proportional to the initial Euclidean distance between source and target fossil taxa. This standardization accounts for the expectation that source populations initially located closer to the target would require fewer generations in the simulations. The standardized values were used to compare the mean number of generations between different evolutionary scenarios. As mentioned previously, the mean number of generations was used as a proxy for the macroevolutionary evolvability of fossil taxa, as it reflects the ability to respond rapidly to repeated generation‐to‐generation selection. To assess the reliability of the simulation outcomes, we examined parameter convergence (Figures S2–S8). These figures demonstrate that the mean and variance of the number of generations, as well as the correlation coefficients between outcome variables, asymptotically stabilize with increasing iteration number, supporting the robustness and interpretability of our results.

It is important to emphasize that we are not proposing that these simulation scenarios are plausible or actual evolutionary pathways. Our aim was to test whether the highly derived skull morphology in *Paranthropus* may have lowered their ability to evolve toward a new adaptive peak within the observed morphospace of Plio‐Pleistocene hominins. In this way, A.L. 444‐2 and KNM‐ER 1813 represent convenient points of comparison for OH 5. These simulations were designed to shed light on the long‐term consequences of the derived skull morphology in *Paranthropus* species in the context of feeding biomechanics.

#### 2.3.3 Visualization of Simulation Outcomes

For visualization of the average evolutionary trajectories, principal component analysis (PCA) was conducted using all 29 measurements from *Homo*, *Pan*, and two fossil hominins in the simulations. The grand mean and PC coefficients from the original PCA were used to calculate PC scores for the evolving population of each generation. This ensured that the evolving morphologies were consistently represented within the original morphospace defined by *Pan*, *Homo*, and the two fossil hominins used in the simulations. To allow comparisons across simulation iterations with varying numbers of generations to reach the target, the PC scores of the evolving populations were standardized on a scale from 0% (start of the simulation) to 100% (end of the simulation). The average evolutionary trajectories from 1000 simulation iterations were then plotted within the original PC morphospace using the first three PCs (with PC3 shown in Figure S9). The fossil hominin not included in the simulations was projected into the original PC morphospace for each evolutionary scenario. The original PC coefficients for the first three axes of the extant taxa and the two fossil hominins used in the simulations are presented in Table S4.

Furthermore, the Euclidean morphological distances to the target throughout the simulations were expressed as percentages relative to the initial distances between fossil hominins. To allow comparisons across simulation iterations with varying numbers of generations to reach the target, the morphological distances of the evolving populations to the target were standardized on a scale from 0% (start of the simulation) to 100% (end of the simulation).

#### 2.3.4 Evolutionary Parameters in Population V/CV Matrices

Several evolutionary quantitative genetics measures can provide insights into how evolutionary properties in V/CV matrices may facilitate or constrain evolution among the three hominin species (e.g., Hansen and Houle 2008).

##### 2.3.4.1 Evolvability and Conditional Evolvability

The correlation between the vector of multivariate differences in means for each pair of extinct hominin species (Δz) and the normalized PC1 eigenvector of the chimpanzee or human V/CV matrices provides information about how well species differences are aligned with PC1 (see Table S3). This is most informative when PC1 (direction of greatest phenotypic variance; p_max_) accounts for a disproportionately large amount of variation. The correlation between p_max_ and the Δz vector is identical but of opposite sign when the latter is calculated as the difference from species A to B or species B to A.

Hansen and Houle's (2008) evolvability parameters (unconditional evolvability and conditional evolvability; *e* and *c*) provide insights into how the V/CV structure more broadly might facilitate or hinder evolution in a particular direction. Specifically, *e* and *c* measure whether Δz aligns with a direction of high evolvability, or variance, in the V/CV matrix. These measures do not just consider p_max_, but also incorporate other dimensions of relatively high variance and are particularly useful when variance is spread more equitably across multiple dimensions.

These evolutionary parameters (*e* and *c*; Hansen and Houle 2008) are derived from the breeder's equation (or Lande equation): Δz = Gβ, where Δz is the evolutionary change in a vector of trait means as mentioned above, β is the directional selection gradient, and G is the additive genetic V/CV matrix (Lande 1979). We substitute the phenotypic V/CV matrix (P) for G, which is appropriate if the matrices are equal or proportional within populations (Cheverud 1988). Rearranging the breeder's equation and substituting P for G clarifies that β can be calculated as P^−1^Δz. Accordingly, *e* is calculated as follows: $e\left(\beta \right)\equiv \frac{\beta^{\prime }P\beta }{{\left|\beta \right|}^{2}},$ where β and P are defined above and 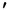 denotes a matrix transpose (note that $e\left(\beta \right)\equiv \beta^{\prime }P\beta$ since β is normalized and thus has unit length, $\left|\beta \right|=1$) (Hansen and Houle 2008). As illustrated in fig. 1 of Hansen and Houle (2008), *e* is the length of the projection of Δz in the direction of β in morphospace. This reflects the extent to which variance in the V/CV matrix aligns with the direction of evolutionary change between two fossil species in the current study. The related measure *c* is calculated as follows: $c\left(\beta \right)={\left(\beta^{\prime }P^{-1}\beta \right)}^{-1}$, where β is the normalized directional selection gradient (Hansen and Houle 2008). The parameter *c* is the length of the response vector in the direction of β when the population is constrained to evolve only in the direction of β. Thus, *c* reflects the reduction in (unconditional) evolvability of β after accounting for conditioning on other traits. We evaluated whether the evolutionary statistics (*e* and *c*, as well as the correlation coefficient with p_max_) differed significantly when using *H. sapiens* vs. *P. troglodytes* V/CV patterns as follows: (1) specimens were resampled with replacement (i.e., bootstrapped) from each extant taxon equal to the original sample size of that species; (2) *e* and *c* values of β, as well as the correlation coefficient with p_max_ were calculated for a pair of fossil hominins; (3) Step 2 was repeated 999 times for each *H. sapiens* or *P. troglodytes* V/CV matrix; and (4) significance was assessed by testing whether the observed values (*e*, *c*, correlation coefficient) fell outside the 2.5–97.5th percentile range of the bootstrapped distribution of the comparison taxon (*α* = 0.05).

In addition to *e* and *c*, we calculated mean evolvability ($\bar{e}$) and conditional evolvability ($\bar{c}$) for both extant and estimated V/CV matrices. Mean evolvability is defined as $\bar{e}\equiv E\left[\beta^{\prime }P\beta \right]$, where β is a normalized selection gradient and P is the phenotypic V/CV matrix (Hansen and Houle 2008). This measure represents the average magnitude of the evolutionary response in the direction of selection for a given P matrix, averaged over a large number of randomly oriented selection vectors. Accordingly, $\bar{e}$ is closely related to the overall amount of variation in P and can be approximated by $E\left[\text{λ}\right]$, the average eigenvalue of P. Mean conditional evolvability is calculated as $\bar{c}\equiv E\left[{\left(\beta^{\prime }P^{-1}\beta \right)}^{-1}\right]$ (Hansen and Houle 2008). This represents the average response in the direction of β, assuming that evolutionary change is constrained to that direction across many possible selection vectors. In other words, $\bar{c}$ reflects the expected evolutionary change in a trait under the condition that the strength of stabilizing selection on other traits is in balance with the strength of directional selection on the focal trait. We calculated $\bar{c}$ by averaging the values obtained from 1000 randomly generated, normalized selection gradients (β), following the method outlined by Hansen and Houle (2008).

In the current study, size correction was not applied to the data as size is a biologically meaningful factor and an integral component of evolutionary processes in viability selection modeling (or morphological evolution more broadly) (see Rolian (2020) for a similar approach). Accordingly, the evolutionary parameters (e.g., *e* and *c*) in the V/CV matrices were also calculated without size correction to maintain compatibility with the simulation outcomes. We acknowledge, however, the potential pitfalls of comparing (conditional) evolvability between V/CV matrices without size correction (Hansen and Houle 2008; Hansen et al. 2024). Mean scaling, for example, is often necessary to standardize traits with differing units, allowing evolutionary changes to be interpreted and compared as proportions relative to trait means. However, it should be noted that all traits in this study were measured in the same units (i.e., millimeters), and that OH 5 and A.L. 444‐2 exhibited similar dimensions across many traits with neither specimen being consistently larger than the other (geometric means: 23.7 for KNM‐ER 1813, 34.5 for A.L. 444‐2, and 35.7 for OH 5). Moreover, mean‐scaled evolvability may still be related to trait means in general for several biological reasons (see Hansen et al. (2024) for review). Nevertheless, it is worth emphasizing that the results concerning (conditional) evolvability in the V/CV matrices in the present study should be interpreted with caution, and specifically within the context of their comparison with the simulation outcomes.

Additionally, adjusting the P matrix for noise, as suggested by Marroig et al. (2012), can be important when variance considered “insignificant” may actually represent noise that affects the outcomes of the study. However, we did not apply this adjustment because the measurements in the current study vary greatly in scale—for instance, zygomatic arch vs. temporal muscle size measurements. In such cases, there is a risk that the noise adjustment method of Marroig et al. (2012), which excludes higher PCs with “insignificant” eigenvalues, may inadvertently eliminate meaningful information from variables with smaller magnitudes. For example, variance in large cranial measurements considered noise may be difficult to distinguish from variance in smaller measurements when represented together as eigenvalues in higher PCs. Therefore, while this type of adjustment may be appropriate when using landmark data or linear distances with similar scales, we believe it is not suitable for our dataset. In other words, when measurements vary widely in scale, identifying which variances are “insignificant” becomes arbitrary and potentially misleading.

##### 2.3.4.2 Mean Trait‐Wise Autonomy and Integration

Trait‐wise autonomy (*a*) is defined as the proportion of a trait's phenotypic variance that is unconstrained by covariation with other traits. This *a* parameter is calculated as ${\left({\left[P^{-1}\right]}_{jj}{\left[P\right]}_{jj}\right)}^{-1}$, where ${\left[P\right]}_{jj}$ signifies the *jj*th element of the P matrix (Hansen and Houle 2008). This formulation represents a nondirectional measure, as it does not involve a selection vector. Each trait has its own *a* value, and the mean across all traits was calculated from the V/CV matrix at each generation. Thus, mean trait‐wise autonomy (mean *a*) is not the same as mean autonomy ($\bar{a}$), which represents the degree of response averaged over many directions (e.g., 1000 random selection vectors) in phenotypic space (sensu Hansen and Houle 2008). A lower mean *a* implies that a greater portion of traits' evolvability is constrained by covariation with other traits, thereby reducing their independence. In other words, *a* can also be defined as the ratio of *c* to *e*: $a\equiv \frac{c}{e}$ (Hansen and Houle 2008).

Integration is measured as the variance of eigenvalues (VE) of the correlation matrix (Wagner 1990; Pavlicev et al. 2009; Rolian 2020). Thus, higher values of VE (i.e., a higher variance among eigenvalues) suggest stronger integration, as more variance is concentrated along fewer dimensions. Conversely, lower VE values indicate that variation is more evenly distributed across multiple dimensions. Mean *a* and VE were calculated for the extant V/CV matrices but not for the estimated V/CV matrices (i.e., VCV_e_), as both measures are scale‐independent (see above), resulting in identical values for the corresponding estimated V/CV matrices.

Throughout the simulation process, we recorded mean *a* and VE for the evolving populations. Both mean *a* and VE are scale‐independent and nondirectional measures (cf. $\bar{e}$ and $\bar{c}$), making them well‐suited for examining the evolutionary properties of evolving V/CV matrices in the simulations. For example, interpreting the mean values of response vectors to selection vectors can be problematic when the underlying distribution is bimodal, as intermediate mean values may not accurately reflect the true structure of the response (Rohlf 2017). In viability selection modeling, this issue is particularly challenging to resolve due to the presence of hundreds or even thousands of V/CV matrices generated across numerous generations at each iteration. To allow comparisons across simulation iterations with varying numbers of generations to reach the target, both the mean *a* and VE of the evolving populations were standardized on a scale from 0% (start of the simulation) to 100% (end of the simulation).

Within each simulation iteration, the average values of mean *a* and VE were calculated, and Spearman's rank correlation coefficients were then computed with the number of generations required to reach the target across 1000 simulation iterations for each evolutionary scenario (i.e., 1000 values for each statistic per evolutionary scenario). Spearman's rank correlation coefficient was used as the distribution of the number of generations in each scenario violated normality assumptions according to the Shapiro–Wilk test (*p* < 0.05). This analysis aimed to infer the relationship between the number of generations to reach the target and the evolutionary properties of the evolving V/CV matrices in the simulations.

Within each simulation iteration, Pearson correlation coefficients were calculated both between mean *a* and VE, and between mean *a* or VE and the percentage of morphological distance to the target on a scale from 0% (start of the simulation) to 100% (end of the simulation). For this, we computed the changes in the percentage of morphological distance, resulting in 99 differences per simulation iteration, which represent changes to the distance from the evolving to target population throughout the simulation. As there is no morphological change after the final simulation iteration, we excluded the final values of mean *a* and VE corresponding to that iteration from the correlation calculations. This process yielded 1000 coefficients for each correlation analysis under each evolutionary scenario, from which the mean and standard deviation were subsequently calculated.

## 3 Results

### 3.1 Simulation Outcomes

**FIGURE 3:**
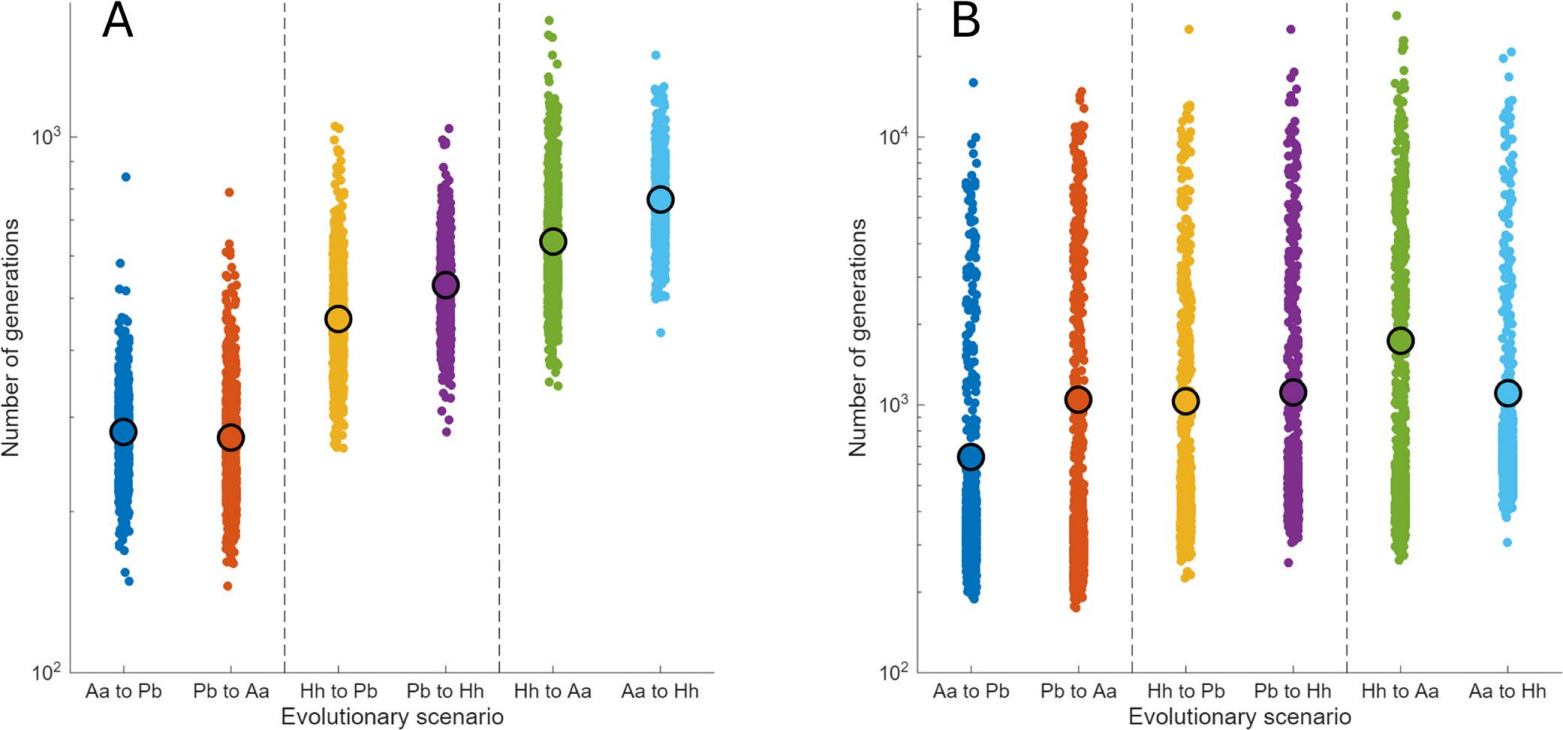
Number of generations (log scale) required to reach new adaptive peaks between fossil hominin pairs. Simulations were conducted using the estimated variance/covariance (V/CV) matrix derived from trait V/CV pattern in (A) humans and (B) chimpanzees. Note that the *y*‐axis scales are not the same in panels (A and B). Each point represents the number of generations for a single simulation iteration, and larger circled dots of the corresponding color indicate the mean across 1000 iterations. Aa = *Australopithecus afarensis* (A.L. 444‐2); Hh = *Homo habilis* (KNM‐ER 1813); Pb = *Paranthropus boisei* (OH 5).

On average, the mean number of generations to simulate evolution from OH 5 (*P. boisei*) to KNM‐ER 1813 ( *H. habilis* ) was larger than the reverse direction when either the human or chimpanzee V/CV patterns were used (Table 2; Figure 3). However, when using the chimpanzee V/CV pattern, the 95% confidence intervals for the two directions showed considerable overlap, suggesting that the difference between directions is less distinct under this model. The mean number of generations from OH 5 to A.L. 444‐2 (*Au. afarensis*) was, on average, larger than the reverse direction when using the chimpanzee V/CV patterns, whereas the opposite result was observed when using human V/CV patterns (Table 2; Figure 3).

**TABLE 2:** The outcomes of 1000 viability selection simulations.

Evolutionary scenario	Initial Euclidean distance	Estimated variance/covariance (V/CV) matrix					
		Human‐like V/CV pattern			Chimpanzee‐like V/CV pattern		
		Mean number of generations	95% CI of number of generations^a^	Standardized values^b^	Mean number of generations	95% CI of number of generations	Standardized values
Aa to Pb	39.96	281 (195–411)^c^	281 ± 3.48	7.0 (4.9–10.2)	**639** ^d^ **(239–4238)**	**639 ± 72.19**	**16.0 (6.0–106.1)**
Pb to Aa		275 (184–439)	275 ± 4.19	6.9 (4.6–11.0)	**1043 (213–7525)**	**1043 ± 120.66**	**26.1 (5.3–188.3)**
Hh to Pb	116.77	**457 (310–705)**	**457 ± 6.46**	**3.9 (2.7–6.0)**	**1032 (285–7433)**	**1032 ± 118.23**	**8.8 (2.4–63.7)**
Pb to Hh		**530 (372–741)**	**530 ± 5.93**	**4.5 (3.2–6.3)**	**1117 (350–7772)**	**1117 ± 129.52**	**9.5 (3.0–66.6)**
Hh to Aa	134.37	639 (425–1076)	639 ± 10.18	4.8 (3.2–8.0)	1739 (338–10,998)	1739 ± 186.38	12.9 (2.5–81.8)
Aa to Hh		765 (546–1032)	765 ± 7.76	5.7 (4.1–7.7)	1107 (448–7257)	1107 ± 120.25	8.2 (3.3–54.0)

Abbreviations: Aa = *Australopithecus afarensis* (A.L. 444‐2); Hh = *Homo habilis* (KNM‐ER 1813); Pb = *Paranthropus boisei* (OH 5).

^a^ 95% confidence interval (CI) = mean ± 1.96 × standard error.

^b^ Proportional to the initial Euclidean distance.

^c^ 2.5–97.5th percentile range of the number of generations from 1000 simulations in parentheses.

^d^ Results that align with our prediction are bolded.

We also compared evolution between *Au. afarensis* and *H. habilis*, although this does not directly test our hypothesis. On average, the evolutionary change from A.L. 444‐2 to KNM‐ER 1813 took fewer generations when using the chimpanzee V/CV pattern than the reverse direction, whereas the opposite result was observed when using the human V/CV pattern.

In standardized values, the mean number of generations was, on average, larger in the simulations between OH 5 and A.L. 444‐2 than in those between the OH 5 and KNM‐ER 1813 or between KNM‐ER 1813 and A.L. 444‐2, regardless of the direction of simulated evolution or V/CV patterns of extant taxa (Table 2).

**FIGURE 4:**
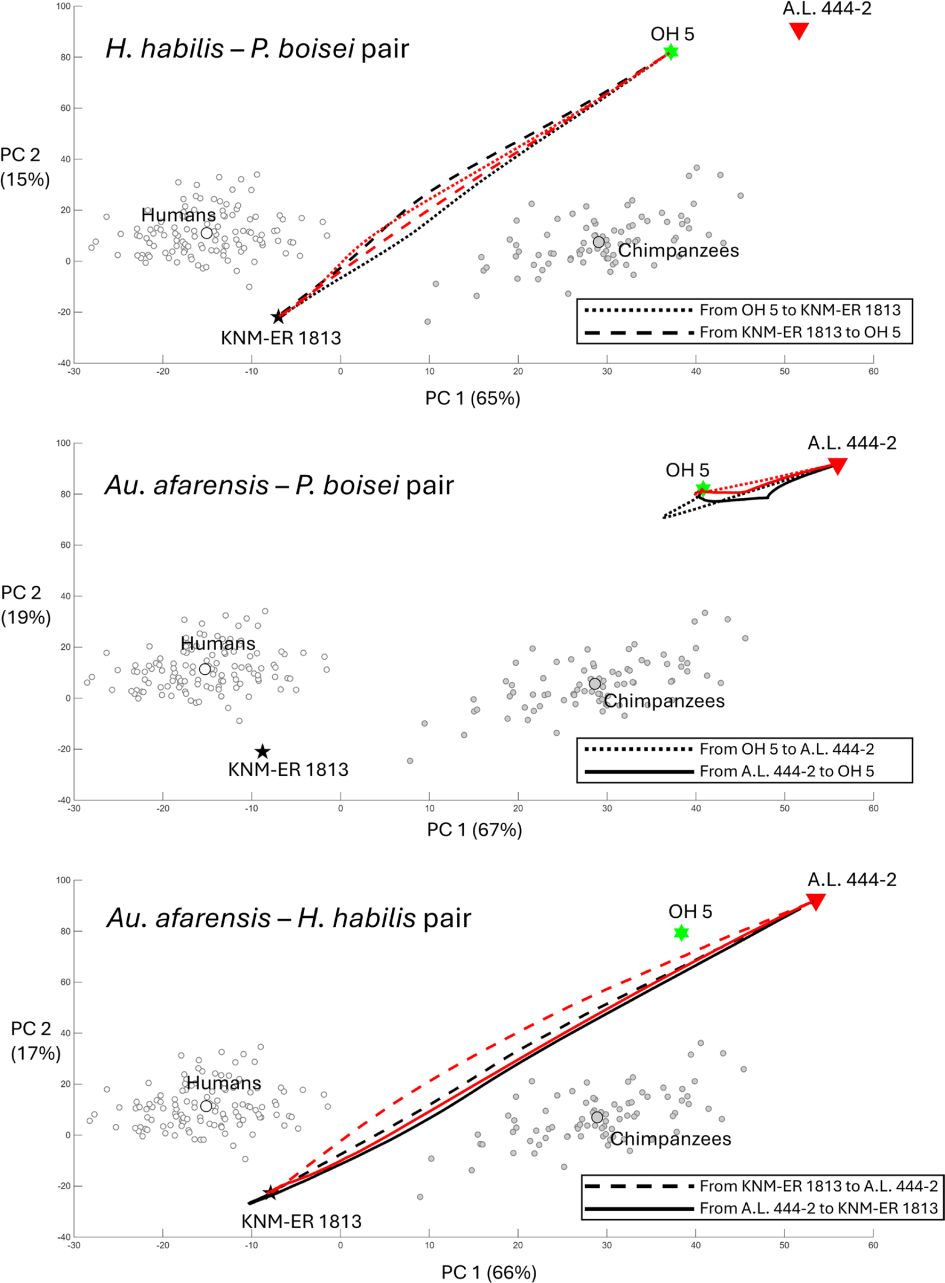
Scatter plots of the first two principal components (PCs) of extant taxa and fossil hominins, along with the average evolutionary trajectories from the simulations. PC plots are generated using data from *Homo sapiens* , *Pan troglodytes* , and two fossil hominins included in the simulations. The fossil hominin not included in the simulations is later projected into the PC morphospace. White and gray circles represent *H. sapiens* and *P. troglodytes* specimens, respectively, in PC plots, with the centroids having larger size and less opacity. Black pentagram: KNM‐ER 1813 (*Homo habilis*); Green hexagram: OH 5 (*Paranthropus boisei*); Red triangle: A.L. 444‐2 (*Australopithecus afarensis*); Red lines: Evolutionary trajectories based on the estimated variance/covariance (V/CV) matrix using human trait V/CV pattern; Black lines: Evolutionary trajectories based on the estimated V/CV matrix using chimpanzee trait V/CV pattern; Solid lines: Trajectories from *Au. afarensis* to other hominins; Dotted lines: Trajectories from *P. boisei* to other hominins; Dashed lines: Trajectories from *H. habilis* to other hominins.

On the PC 1 and 2 axes, the average evolutionary trajectories generally follow straightforward paths, with slight to moderate curvature during the middle phases of the simulations (Figure 4). One exception was the trajectory from A.L. 444‐2 to KNM‐ER 1813 based on the estimated V/CV matrix using chimpanzee‐like patterns. In this case, the evolving population reached the target after an extended path. Nevertheless, it required fewer generations than the reverse direction (1107 vs. 1739 mean generations; Table 2). Another interesting case was the average trajectory from OH 5 to A.L. 444‐2, also based on chimpanzee‐like patterns. Here, the evolving population initially moved in the opposite direction from the target before correcting course and ultimately reaching it (Figure 4). Notably, this scenario took more generations to complete than the reverse direction (1043 vs. 639 mean generations; Table 2).

**FIGURE 5:**
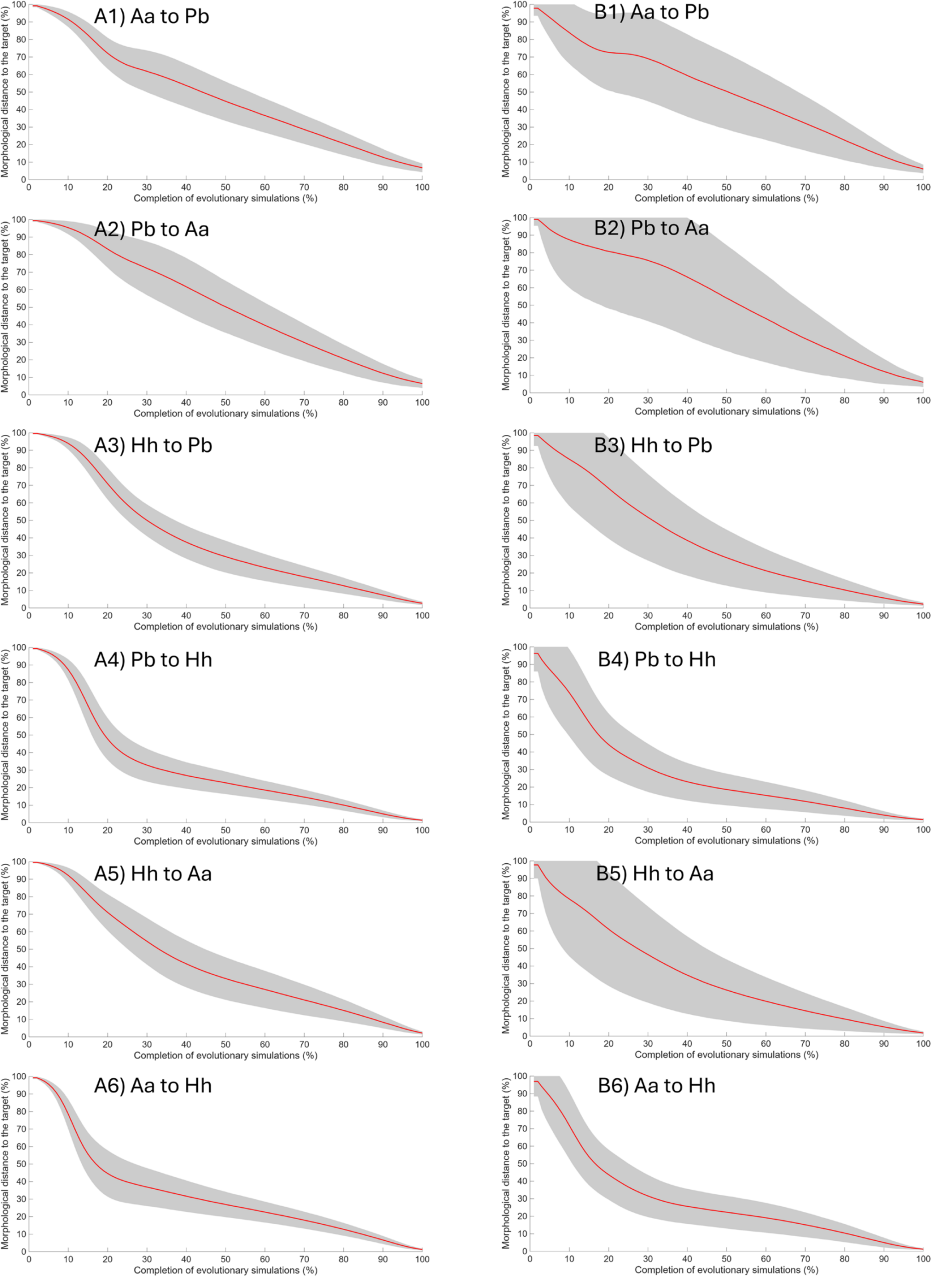
Euclidean morphological distance throughout the simulation process. For each evolutionary scenario, the morphological distance (%) of the evolving population to the target was calculated using the estimated variance/covariance (V/CV) matrix derived from trait V/CV pattern in (A) humans and (B) chimpanzees. The red line represents the average of 1000 simulation iterations, scaled from 0% (start of the simulation) to 100% (end of the simulation). The gray area indicates the standard deviation. Aa = *Australopithecus afarensis* (A.L. 444‐2); Hh = *Homo habilis* (KNM‐ER 1813); Pb = *Paranthropus boisei* (OH 5).

In comparison to other pairs of hominins, the OH 5–A.L. 444‐2 pair showed more gradual morphological changes overall, with occasional periods of stasis (Figure 5). Furthermore, the degree of morphological changes was slower in the early phases (approximately during 20%–30% of the trajectory) of the evolutionary scenario from OH 5 to A.L. 444‐2 compared to the reverse direction. In contrast, the opposite trend was observed in the scenario from OH 5 or A.L. 444‐2 to KNM‐ER 1813. For both the OH 5–KNM‐ER 1813 and A.L. 444‐2–KNM‐ER 1813 pairs, morphological distances to the target decreased more rapidly during the early phases of the simulations, followed by more gradual changes (Figure 5). In all cases, the standard deviation appears to be smaller when using human‐like V/CV patterns compared to chimpanzee‐like patterns.

### 3.2 The Evolutionary Parameters in Population V/CV Matrices

#### 3.2.1 Evolvability and Conditional Evolvability

Evolvability (*e*) in the A.L. 444‐2–KNM‐ER 1813 pair was significantly larger when modeled with the *H. sapiens* V/CV matrix than with the *P. troglodytes* V/CV matrix (*p* < 0.05). Conditional evolvability (*c*) for each pair of fossil hominins was significantly larger with the *H. sapiens* V/CV matrix than with the *P. troglodytes* V/CV matrix (*p* < 0.05) (Table 3).

**TABLE 3:** Evolvability and conditional evolvability of the directional selection gradient (β) between fossil hominins based on extant taxon variance/covariance (V/CV) matrix.

Evolutionary scenario	Evolvability (*e*)		Conditional evolvability (*c*)	
	*Homo sapiens* V/CV matrix	*Pan troglodytes* V/CV matrix	*Homo sapiens* V/CV matrix	*Pan troglodytes* V/CV matrix
Pb and Aa	0.50 (0.38–0.63)^a^	0.49 (0.34–0.64)	**0.20 (0.10–0.20)**	0.15 (0.05–0.13)
Pb and Hh	0.59 (0.44–0.76)	0.60 (0.41–0.77)	**0.27 (0.14–0.26)**	**0.11 (0.03–0.09)**
Aa and Hh	**0.77 (0.57–0.99)**	**0.39 (0.28–0.49)**	**0.33 (0.17–0.32)**	**0.08 (0.02–0.07)**

Abbreviations: Aa = *Australopithecus afarensis* (A.L. 444‐2); Hh = *Homo habilis* (KNM‐ER 1813); Pb = *Paranthropus boisei* (OH 5).

^a^ Observed evolvability or conditional evolvability and 2.5–97.5th percentile range from 1000 bootstrapping procedures in parentheses. Observed values that fall outside the 2.5–97.5th percentile range of the comparison taxon are shown in bold.

The *e* and *c* values were smallest for the OH 5–A.L. 444‐2 pair, followed by the OH 5–KNM‐ER 1813 pair and the A.L. 444‐2–KNM‐ER 1813 pair based on the *H. sapiens* V/CV matrix. Using the *P. troglodytes* V/CV matrix, the opposite trend was found in *c* results, as the A.L. 444‐2–KNM‐ER 1813 pair exhibited the smallest *c* value, followed by the OH 5–KNM‐ER 1813 pair and the OH 5–A.L. 444‐2 pair.

For the V/CV matrices of extant taxa, $\bar{e}$ was higher in chimpanzees than in humans, whereas $\bar{c}$ was higher in humans. Similarly, the estimated V/CV matrices of fossil hominins showed higher $\bar{c}$ values when based on a human‐like pattern, while $\bar{e}$ was higher when based on a chimpanzee‐like pattern (Table 4). Among the estimated V/CV matrices, $\bar{e}$ was consistently highest in *Au. afarensis*, followed by *P. boisei*, and lowest in *H. habilis* . In contrast, $\bar{c}$ was always highest in *P. boisei*, followed by *Au. afarensis*, and lowest in *H. habilis* .

**TABLE 4:** Mean evolvability ($\bar{e}$) and mean conditional evolvability ($\bar{c}$) of the extant and estimated variance/covariance (V/CV) matrices.

Index	Extant taxa V/CV matrix		Estimated V/CV matrix					
			Human‐like V/CV pattern			Chimpanzee‐like V/CV pattern		
	Humans	Chimpanzees	Pb	Aa	Hh	Pb	Aa	Hh
$\bar{e}$	7.68	10.94	10.15	10.53	6.37	12.28	12.64	8.11
$\bar{c}$	0.73	0.71	1.37	1.28	0.74	1.13	1.04	0.64

Abbreviations: Aa = *Australopithecus afarensis* (A.L. 444‐2); Hh = *Homo habilis* (KNM‐ER 1813); Pb = *Paranthropus boisei* (OH 5).

#### 3.2.2 Mean Trait‐Wise Autonomy and Integration

Mean *a* and VE were 0.34 and 1.54 in the human V/CV matrix, respectively, and 0.24 and 2.64 in the chimpanzee V/CV matrix, respectively.

**FIGURE 6:**
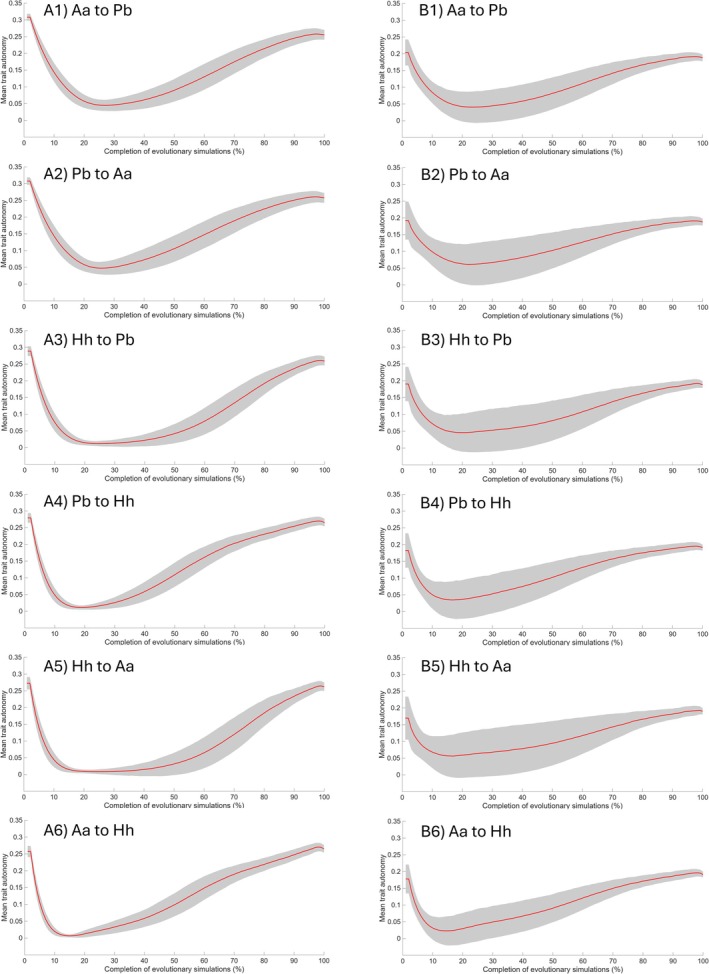
Mean trait‐wise autonomy (*a*) throughout the simulation process. For each evolutionary scenario, the mean *a* of the evolving population was calculated using the estimated variance/covariance (V/CV) matrix derived from trait V/CV pattern in (A) humans and (B) chimpanzees. The red line represents the average of 1000 simulation iterations, scaled from 0% (start of the simulation) to 100% (end of the simulation). The gray area indicates the standard deviation. Aa = *Australopithecus afarensis* (A.L. 444‐2); Hh = *Homo habilis* (KNM‐ER 1813); Pb = *Paranthropus boisei* (OH 5).

**FIGURE 7:**
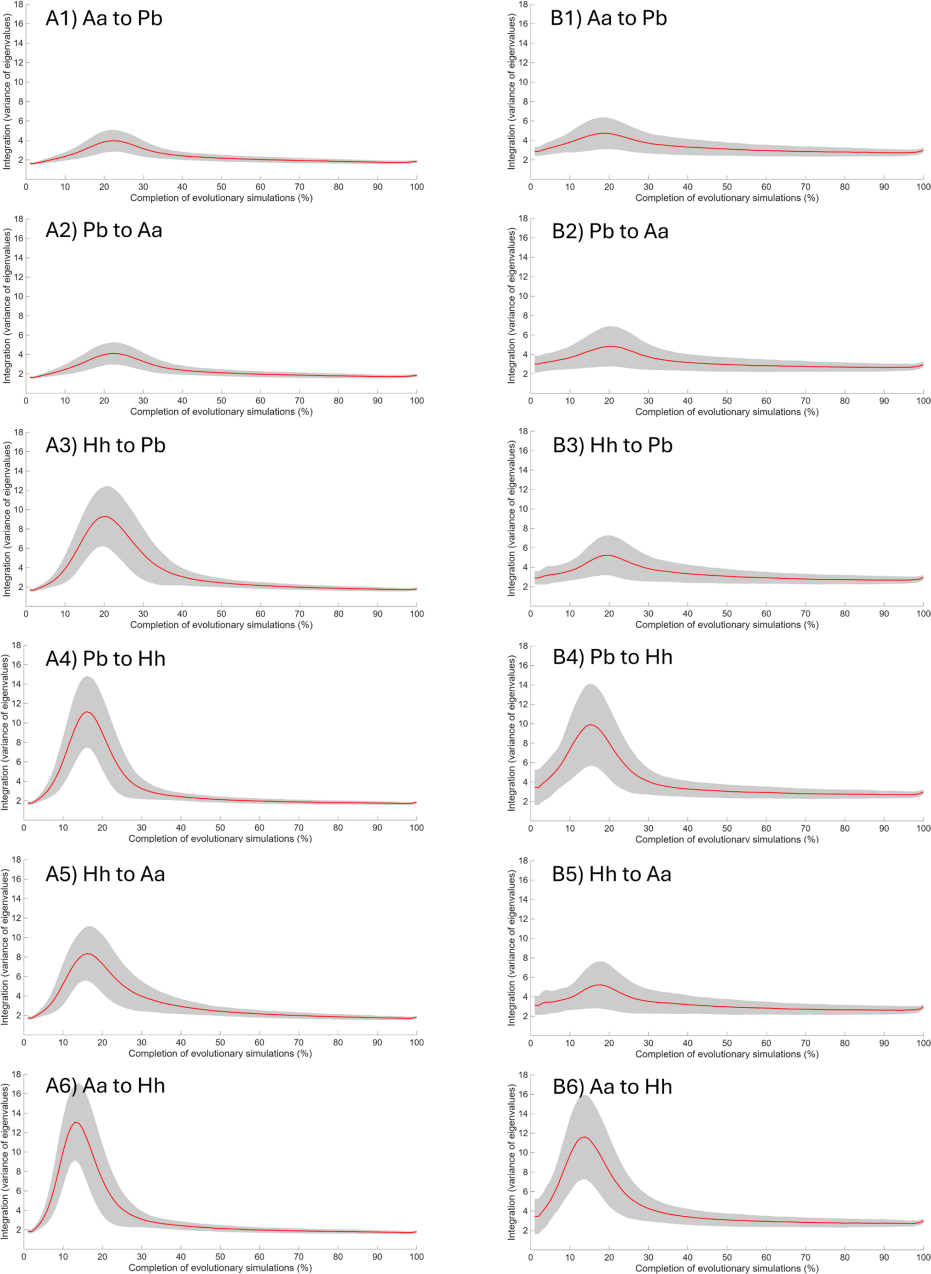
Integration (variance of eigenvalues; VE) throughout the simulation process. For each evolutionary scenario, VE of the evolving population was calculated using the estimated variance/covariance (V/CV) matrix derived from trait V/CV pattern in (A) humans and (B) chimpanzees. The red line represents the average of 1000 simulation iterations, scaled from 0% (start of the simulation) to 100% (end of the simulation). The gray area indicates the standard deviation. Aa = *Australopithecus afarensis* (A.L. 444‐2); Hh = *Homo habilis* (KNM‐ER 1813); Pb = *Paranthropus boisei* (OH 5).

Across 1000 simulation iterations, mean *a* was slightly higher, with a smaller standard deviation, when the estimated V/CV matrix was derived from human‐like V/CV patterns compared to those based on chimpanzee‐like patterns (Figure 6). In all cases, the mean *a* declined during the early phases of the simulation (approximately during 20%–30% of the trajectory) and recovered as the population approached the target. Integration (VE) began at a higher value when the estimated V/CV matrices were based on chimpanzee‐like patterns compared to those derived from human‐like patterns (Figure 7). However, VE increased more rapidly and reached higher values during the early phases when the estimated V/CV matrices were based on human‐like patterns before subsequently declining. Furthermore, VE was relatively higher in the early phases of simulations directed toward *H. habilis* compared to the reverse direction.

In the estimated V/CV matrices derived from chimpanzee‐like V/CV patterns, the number of generations was positively correlated with the average values of mean *a* and negatively correlated with the average values of VE (Table 5). With human‐like patterns, the number of generations was negatively correlated with the average values of both mean *a* and VE in most cases (Table 5). In the correlation analysis conducted within each simulation iteration, mean *a* and VE exhibited moderate‐to‐strong negative correlations (Table 6). The degree of morphological changes was negatively correlated with mean *a* but positively correlated with VE, except for the OH 5–A.L. 444‐2 pair. Additionally, the mean of the 1000 correlation coefficients between the degree of morphological changes and mean *a* or VE was relatively lower for the OH 5–A.L. 444‐2 pair than for other pairs.

**TABLE 5:** The Spearman's rank correlation coefficients between the average values of mean trait‐wise autonomy (*a*) or integration (variance of eigenvalues; VE) of evolving populations and the number of generations required to reach the target in the simulations. The average value of mean *a* and VE, as well as the number of generations, were calculated from each simulation iteration (i.e., 1000 values for each statistic per evolutionary scenario).

Evolutionary scenario	Estimated variance/covariance (V/CV) matrix			
	Human‐like V/CV pattern		Chimpanzee‐like V/CV pattern	
	Average of mean *a*	Average of VE	Average of mean *a*	Average of VE
Aa to Pb	−0.20^a^	0.11	0.19	−0.19
Pb to Aa	−0.02	−0.09	0.52	−0.52
Hh to Pb	−0.25	−0.24	0.48	−0.56
Pb to Hh	−0.21	−0.09	0.38	−0.40
Hh to Aa	−0.22	−0.26	0.64	−0.67
Aa to Hh	−0.36	−0.14	0.20	−0.24

Abbreviations: Aa = *Australopithecus afarensis* (A.L. 444‐2); Hh = *Homo habilis* (KNM‐ER 1813); Pb = *Paranthropus boisei* (OH 5).

^a^ Correlation coefficients between each statistic and the number of generations across 1000 simulations.

**TABLE 6:** The Pearson correlation coefficients between the degree of morphological changes (Morph) and mean trait‐wise autonomy (*a*) or integration (variance of eigenvalues; VE) within each simulation iteration (i.e., 1000 correlation coefficients). The final values of mean *a* and VE (corresponding to 100% of each simulation iteration) were excluded from the correlation analyses, as each simulation iteration contained 99 sequential differences in the percentage of morphological distance to the target.

Evolutionary scenario	Estimated variance/covariance (V/CV) matrix					
	Human‐like V/CV pattern			Chimpanzee‐like V/CV pattern		
	Morph vs. Mean *a*	Morph vs. VE	Mean *a* vs. VE	Morph vs. Mean *a*	Morph vs. VE	Mean *a* vs. VE
Aa to Pb	−0.15 (0.17)^a^	0.16 (0.24)	−0.72 (0.09)	−0.01 (0.21)	−0.08 (0.23)	−0.62 (0.19)
Pb to Aa	−0.17 (0.19)	0.12 (0.26)	−0.73 (0.08)	−0.07 (0.25)	−0.09 (0.21)	−0.67 (0.14)
Hh to Pb	−0.44 (0.15)	0.71 (0.13)	−0.58 (0.07)	−0.53 (0.17)	0.49 (0.19)	−0.64 (0.14)
Pb to Hh	−0.47 (0.10)	0.80 (0.13)	−0.62 (0.06)	−0.52 (0.12)	0.71 (0.11)	−0.67 (0.08)
Hh to Aa	−0.33 (0.20)	0.52 (0.25)	−0.54 (0.08)	−0.52 (0.18)	0.46 (0.21)	−0.64 (0.13)
Aa to Hh	−0.36 (0.12)	0.67 (0.19)	−0.61 (0.06)	−0.43 (0.13)	0.67 (0.13)	−0.66 (0.08)

Abbreviations: Aa = *Australopithecus afarensis* (A.L. 444‐2); Hh = *Homo habilis* (KNM‐ER 1813); Pb = *Paranthropus boisei* (OH 5).

^a^ Mean of 1000 correlation coefficients with standard deviation in parentheses.

## 4 Discussion

The primary focus of this study was to investigate the potential evolutionary consequences of the highly derived cranial morphology in *Paranthropus*. We hypothesized that the highly derived cranial features related to feeding biomechanics observed in *Paranthropus* may have played a significant role in reducing their macroevolutionary evolvability.

### 4.1 Hypothesis Evaluation

The results aligned with the prediction that *P. boisei* would, on average, require a larger number of generations to reach the *H. habilis* cranial morphotype than *H. habilis* did to reach the *P. boisei* morphotype, regardless of whether the human or chimpanzee patterns of population V/CV were used (i.e., the estimated V/CV matrix of a fossil taxon using the extant taxon trait V/CV pattern). Likewise, it took longer for *P. boisei* to evolve toward the *Au. afarensis* morphotype, but only when chimpanzee‐like patterns were used to estimate the *P. boisei* (co)variance structure. In contrast, it took longer for *Au. afarensis* to evolve toward the *P. boisei* adaptive peak when the human pattern of trait variances and covariances was used. Together, these results provide partial support for the hypothesis as the hypertrophied craniodental morphology of *P. boisei* reduced its macroevolutionary evolvability under certain conditions related to the direction of selection and underlying V/CV pattern. Additionally, the patterns in the degree of evolutionary change (e.g., Figure 5) were similar for each evolutionary scenario, regardless of which extant V/CV pattern was used for the source population, likely reflecting the importance of the initial morphology in shaping the evolutionary trajectories. Thus, given the effect of the extant V/CV pattern on the number of generations, reduced macroevolutionary evolvability may have resulted from both the initial cranial morphology of *P. boisei* and the V/CV structures, as well as the changes in the latter along the evolutionary trajectories (see Section 4.2 for a more detailed discussion on this topic).

The third pair, *Au. afarensis* and *H. habilis* , provides an interesting point of comparison but does not bear directly on our hypothesis. The results for the comparison of *Au. afarensis* and *H. habilis* are dependent on which extant taxon was used as a proxy of fossil phenotypic variance and covariance. While *Au. afarensis* required a longer “evolutionary time” to reach the *H. habilis* morphotypes than in the reverse direction when human variance and covariance data were used to generate populations of fossil taxa, the opposite pattern was observed when the chimpanzee pattern was used. Although there is evidence that some trends seen in *Paranthropus* were expressed to a more limited degree in the “gracile” australopiths (e.g., megadontia), our hypothesis focused on the more derived, “hypertrophied” morphology of *P. boisei*.

### 4.2 Exploring the Relationships Between Evolutionary Parameters and Simulation Outcomes

We found complex (possibly nonlinear) relationships between evolutionary parameters in the population V/CV matrices and macroevolutionary evolvability in fossil hominins. Notably, the results suggest that microevolutionary evolvability in specific directions of selection (*e* and *c*) is generally more predictive of macroevolutionary evolvability in the simulations than measures averaged across a large number of randomly oriented selection vectors ($\bar{e}$ and $\bar{c}$), depending on the extant V/CV structure, as discussed in more detail below.

#### 4.2.1 Discrepancy Between Micro‐ and Macroevolutionary Evolvability

In the case where *P. boisei* evolved toward the *Au. afarensis* morphotype, the average evolutionary trajectory suggests that the *Paranthropus* morphology became temporarily “stuck” in morphospace when using the estimated V/CV matrix derived from chimpanzee‐like patterns (Figure 4). The evolving population initially moved in the opposite direction from the target before correcting course and eventually reaching it. Considering the relatively higher $\bar{c}$ in the estimated V/CV matrices of *P. boisei* (Table 4), these results suggest that high evolutionary potential at a microevolutionary scale may not necessarily translate into high evolvability at a macroevolutionary scale. This may be particularly evident when considering the suboptimal or inefficient evolutionary trajectories that evolving populations may follow in morphospace toward new adaptive peaks.

Larger dimensions are generally associated with greater variances and, by inference, higher microevolutionary evolvability (Table 4; Hansen and Houle 2008). This would predict that evolutionary change should occur more rapidly from larger to smaller forms than the reverse. However, our results do not support this expectation. For example, *H. habilis* , which has the smallest overall dimensions and lowest $\bar{e}$ and $\bar{c}$ for the estimated V/CV matrices, was either not markedly different from or evolved more rapidly toward *Au. afarensis* and *P. boisei*—except in the case where *H. habilis* evolved toward *Au. afarensis* using the estimated V/CV matrix based on the chimpanzee‐like patterns. This suggests that *H. habilis* (or early *Homo* more generally) possessed a masticatory apparatus with relatively high macroevolutionary evolvability in certain directions, despite its small size (geometric means: 23.7 for KNM‐ER 1813, 34.5 for A.L. 444‐2, and 35.7 for OH 5) and lower microevolutionary evolvability in the present study (Table 4).

#### 4.2.2 Concordance Between Micro‐ and Macroevolutionary Evolvability

The results showed that *P. boisei* and *Au. afarensis* required relatively longer evolutionary time (i.e., standardized mean number of generations) to reach each other than either did to reach the adaptive peak of *H. habilis* (Figure 4). There is theoretical and empirical evidence that evolutionary changes can occur more rapidly along a line of least evolutionary resistance, which is typically aligned with the axis of greatest genetic or phenotypic (co)variation in morphospace (e.g., g_max_ or p_max_; Schluter 1996; Marroig and Cheverud 2004). Thus, the larger number of standardized generations between *P. boisei* and *Au. afarensis* may reflect the generally lower *e* and *c* values for the *P. boisei–Au. afarensis* pair compared to the *P. boisei–H. habilis* pair (Table 3). Thus, evolutionary change may be slower because of the differences between *P. boisei* and *Au. afarensis* generally do not align with dimensions of high variability (additionally, the *P. boisei*–*Au. afarensis* pair also showed a lower correlation between Δz and p_max_ of the extant V/CV matrices compared to other pairs of hominins, as shown in Table S3). This result implies that directional selection, rather than stochastic processes, produced cranial evolution from gracile (e.g., *Au. afarensis*) to robust (e.g., *P. boisei*) australopiths. This interpretation is supported by previous evolutionary quantitative genetic research on hominin crania, albeit without a biomechanical focus (Ackermann and Cheverud 2004; Schroeder and Ackermann 2023).

Another interesting observation is that it required about two to four times as many generations to produce the same evolutionary changes based on a chimpanzee‐like pattern of trait V/CV compared to the human‐like pattern (Table 2). These results are consistent with the finding that *c* was 1.3–4.1 times larger with the *H. sapiens* V/CV matrix than the *P. troglodytes* V/CV matrix (Table 3). Moreover, $\bar{c}$ was higher in the *H. sapiens* V/CV matrix than the *P. troglodytes* V/CV matrix, and likewise higher in the estimated V/CV matrices based on human‐like patterns, compared to those based on chimpanzee‐like patterns (Table 4).

Thus, the evolution of biomechanically informative measurements in the simulations occurred more rapidly based on the *H. sapiens* pattern of trait covariances than on the *P. troglodytes* pattern, consistent with evolvability statistics observed at the microevolutionary scale. This suggests that despite the generally conserved nature of morphological integration (e.g., patterns of integration) in the skull among primates broadly and hominoids specifically (Ackermann 2002; de Oliveira et al. 2009; Singh et al. 2012; Jung et al. 2023, 2024), the differences in these patterns can have macroevolutionary consequences. This highlights the significance of the evolution of V/CV structure along phylogenetic branches in hominins, especially when such changes enhance both micro‐ and macroevolutionary evolvability. It is tempting to speculate that the human‐like pattern evolved prior to the emergence of *Paranthropus* and thus facilitated the specializations of the masticatory system, but this cannot be confirmed directly.

#### 4.2.3 Mean Trait‐Wise Autonomy and Integration in Relation to Macroevolutionary Evolvability

The macroevolutionary consequences of population V/CV structures were also observed in the results of mean *a* and VE. The average values of VE in each simulation iteration generally showed a negative correlation with the number of generations using both human‐like and chimpanzee‐like patterns (i.e., stronger integration was associated with shorter evolutionary times). Stronger integration aligned with the direction of selection may facilitate morphological evolution along that trajectory more effectively than weaker integration, even when the overall pattern of integration is the same (Goswami et al. 2014; Rolian 2020). Conversely, misalignment with the direction of selection can impose greater constraints. Thus, the result regarding the VE may reflect the former scenario, suggesting that levels of integration facilitated evolution in fossil hominins in the present study. Similar results were found in previous studies (but see Goswami and Polly (2010) for a differing perspective). Navalón et al. (2020) proposed that tight coupling between beak and craniofacial morphologies in Darwin's finches and Hawaiian honeycreepers may have enabled rapid evolutionary diversification. Likewise, Hedrick et al. (2020) observed greater cranial diversity in phyllostomid bats, potentially facilitated by strong integration.

Consistent with this, greater morphological change within each simulation iteration was generally associated with higher VE, but lower mean *a*, highlighting the role of integration levels in facilitating evolutionary change (Table 6). This may reflect a rapid increase in VE during the early phases of the simulations, when the degree of morphological changes was also relatively high (Figures 5 and 7). This suggests that evolutionary changes initially occurred primarily along dimensions aligned with the direction of selection and characterized by high integration, thereby moving the evolving population closer to the target at a higher rate. Consequently, greater variance may be concentrated along these dimensions, facilitating more rapid change. Over evolutionary time, as mean *a* increases and VE decreases, the variances within the V/CV matrices presumably became more evenly distributed across multiple dimensions, allowing for greater trait autonomy but resulting in slower rates of change.

However, this effect was substantially weaker or absent in the *P. boisei–Au. afarensis* pair (Table 6). As discussed in Section 4.2.2, this pair exhibited generally lower values of *e* and *c* than other pairs. Consequently, morphological changes were more gradual, with occasional stasis (i.e., not facilitated), likely because the evolutionary changes in the *P. boisei–Au. afarensis* pair did not align with the dimensions of high variability.

A distinct pattern was also observed wherein mean *a* and VE differently affected simulation outcomes under human‐like and chimpanzee‐like patterns, highlighting the complex relationship between evolutionary properties in the V/CV matrix and macroevolutionary evolvability. Mean *a* and VE are complementary, as strongly integrated traits tend to exhibit higher variational potential that is constrained by covariation with other traits, resulting in lower mean *a* (Wagner 1990; Pavlicev et al. 2009; Rolian 2020). For example, mean *a* was negatively correlated with the degree of morphological changes within each simulation iteration (Table 6). Nevertheless, the average coefficients between mean *a* and VE did not represent a perfectly linear relationship but fell within a moderate‐to‐strong negative range (−0.58 to −0.73), indicating potential independent variability between mean *a* and VE.

Notably, the average values of mean *a* were positively correlated with the number of generations required to reach the target under chimpanzee‐like patterns (i.e., more generations with higher mean *a*), whereas under human‐like patterns, they tended to be negatively correlated (i.e., fewer generations with higher mean *a*) (Table 5). As discussed in Section 4.2.2, the direction of selection among fossil hominins is generally less aligned with dimensions of high variability under chimpanzee‐like V/CV patterns than under human‐like patterns. Consequently, it took approximately two to four times as many generations to achieve the same evolutionary changes based on chimpanzee‐like V/CV patterns compared to human‐like patterns. In this context, under chimpanzee‐like patterns compared to human‐like ones, it may take considerably longer evolutionary time for an evolving population to develop V/CV structures with an appropriate level of autonomy and sufficiently strong integration, such that their high variability is well aligned with the direction of selection. This aspect may also be reflected in the fact that mean *a* (and morphological distance to the target) exhibited consistently greater variability (i.e., standard deviation) across 1000 simulation iterations during most phases when based on chimpanzee‐like V/CV patterns compared to human‐like patterns (Figures 5 and 6). This, in turn, may contribute to the greater variance in the number of generations—characterized by numerous extreme cases—observed in the evolutionary scenarios (Table 2).

In the case of *Paranthropus*, it is possible that they exhibited stronger functional integration than other extinct hominins, similar to what has been observed in capuchins and certain modern human populations consuming mechanically resistant foods (Makedonska et al. 2012; Noback and Harvati 2015; but see Paschetta et al. 2016). Together with the results of mean *a* and VE, this may suggest that mechanisms of morphological integration could have facilitated or constrained cranial evolution in *Paranthropus*, depending on the V/CV structures and whether integration was well aligned with the direction of selection.

### 4.3 Implications for the Evolutionary History of *Paranthropus boisei*

*Paranthropus boisei* is generally viewed as highly derived with respect to craniodental morphology despite long‐standing disagreements about the degree of dietary specialization (e.g., Robinson 1954, 1963; Wood and Strait 2004; Scott et al. 2005; Cerling et al. 2011; Smith et al. 2015; Wynn et al. 2020). Our study does not directly test whether ecological or dietary specialization was a contributing factor in *Paranthropus* extinction (Wood and Strait 2004; Scott et al. 2005; Cerling et al. 2011; Wynn et al. 2020). However, the derived craniodental morphology that characterizes *Paranthropus* is presumably functionally related to feeding and diet, which in turn may reflect ecological strategies. Yet, regardless of whether *Paranthropus* was ecologically or dietarily generalized (eurytopic or euryphagic) or specialized (stenotopic or stenophagic), the results of the current study hint at reduced macroevolutionary evolvability of *P. boisei* cranial morphology, which may have increased its probability of extinction under certain scenarios related to vegetation or other ecological changes during the Mid‐Pleistocene Transition (MPT; 1.3–0.7 Ma). In other words, *P. boisei*'s highly derived morphology may have positioned it in a region of morphospace—or along evolutionary trajectories—from which evolutionary shifts toward new adaptive peaks were more difficult under certain conditions. Additionally, even in the scenario in which *P. boisei* evolved more quickly toward the *Au. afarensis* adaptive peak when using human‐like V/CV patterns, the fact that gracile australopiths went extinct hundreds of thousands of years before the demise of *Paranthropus* may suggest that such a peak no longer existed under the environmental conditions affecting chronologically later populations of *P. boisei*, further limiting the evolutionary options for these populations. Thus, although we do not know whether *Paranthropus* was ecologically over‐specialized, it may nonetheless have been morphologically “over‐derived” (e.g., a population has evolved into a region of morphospace from which it is difficult to evolve toward new regions). While the concept of being over‐derived may be less intuitive than that of being over‐specialized, it offers a more direct interpretation of our results (see Supplementary S1 for further discussion).

### 4.4 Limitations and Future Directions

Certainly, the argument above is not without its limitations. For example, our tests rely on single exemplary fossils and should be confirmed with additional representatives of these species. Moreover, we cannot directly model the population parameters of extinct hominin species, nor can we determine whether *P. boisei* experienced directional selection toward an adaptive peak that resembled *H. habilis* or *Au. afarensis*. For example, we used estimated V/CV matrices for fossil species in the simulations, which may not accurately represent the true parameters of those extinct hominins; thus, the results could differ if sufficient sample sizes and actual V/CV matrices become available. Yet, given that *Paranthropus* and early *Homo*, at least, were broadly contemporaneous in broadly the same landscapes (e.g., the Turkana Basin in eastern Africa; the Malmani dolomite in southern Africa), it is reasonable to infer that such adaptive peaks (anatomy, physiology, and/or behavior) existed when and where these hominin populations lived.

Furthermore, the current study has limitations in uncovering all the mechanisms underlying the evolution of the V/CV matrix during the simulation processes. In contrast, simulation studies by Jones and colleagues have provided theoretical foundations for understanding how the genetic V/CV matrix (and mutation matrix) evolves under specific conditions such as mutation, selection, drift, and a moving adaptive optimum (Jones et al. 2003, 2004, 2007). However, we were unable to disentangle the causal mechanisms underlying structural changes in the V/CV matrix and their direct effect on the simulation outcomes (see Supplementary S2 for additional, albeit limited, interpretation). This limitation may be partly due to the high number of variables (e.g., 29 traits in the current study vs. 2 traits in Jones et al. 2003) used in this study, which substantially increases the complexity of the V/CV matrices, especially considering that hundreds or even thousands of V/CV matrices were generated across numerous generations in each simulation iteration. Consequently, this limitation may hinder our ability to fully unravel the causal relationships underlying the transition between micro‐ and macroevolutionary processes in the simulations. Future research with proper study designs, including V/CV matrices from more diverse extant taxa, would be valuable to address this issue.

In addition, further studies using more *Paranthropus* (e.g., *P. robustus* ) and other fossil hominin cranial specimens are required before drawing firm conclusions. For example, early *Paranthropus* specimens exhibit less derived cranial morphology (e.g., a more prognathic face) as exemplified in the contrasts between *P. aethiopicus* and the younger *P. boisei* in East Africa, and possibly between early *P. robustus* crania from Drimolen and later *P. robustus* crania from Swartkrans in South Africa (Martin et al. 2021). Thus, evolvability needs to be evaluated more broadly within the *Paranthropus* lineage. It has been suggested (e.g., Walker et al. 1986; Martin et al. 2024) that *P. aethiopicus* is an early member of the *P. boisei* lineage, so it would be interesting to examine how the addition of an intermediate “stage” of evolution between *Au. afarensis* and *P. boisei* affect results.

## 5 Conclusion

The findings of this study provide insights into the evolutionary implications of the highly derived skull morphology in *P. boisei* within the context of feeding biomechanics. The results showed that the mean number of generations required to evolve from *P. boisei* to *H. habilis* was greater than in the reverse direction. Similarly, *P. boisei* exhibited slower evolution toward the *Au. afarensis* adaptive peak when a chimpanzee‐like V/CV pattern was used. Additionally, macroevolutionary potential of the *P. boisei* cranium was generally more predictable from microevolutionary evolvability in specific directions of selection (*e* and *c*) and levels of integration than measures averaged across a large number of randomly oriented selection vectors ($\bar{e}$ and $\bar{c}$). Thus, the results are compatible with our hypothesis that *Paranthropus* may have experienced a significant reduction in their macroevolutionary evolvability under certain conditions—particularly if selection favored a cranial morphology similar to *H. habilis* (i.e., “reduced masticatory”). This observed reduction in macroevolutionary evolvability may be attributable not only to the highly derived cranial morphology of *P. boisei* but also to changes in the V/CV structures along the simulated evolutionary trajectories. This study also highlights that nuanced differences in V/CV matrices can have macroevolutionary consequences and this must be considered carefully when choosing extant proxies for extinct species.

## Author Contributions

**Hyunwoo Jung:** conceptualization, methodology, investigation, formal analysis, visualization, writing – original draft, writing – review and editing, data curation, validation. **Campbell Rolian:** conceptualization, methodology, investigation, supervision, writing – original draft, writing – review and editing, validation. **David S. Strait:** conceptualization, methodology, investigation, supervision, writing – original draft, writing – review and editing, validation. **Karen L. Baab:** conceptualization, methodology, data curation, investigation, supervision, funding acquisition, writing – original draft, writing – review and editing, validation.

## Conflicts of Interest

The authors declare no conflicts of interest.

## Supporting information


**Data S1:** Supporting Information.

## Data Availability

The data that support the findings of this study are available from the corresponding author upon reasonable request.
