# Supplementary material for: Evaluating the Evolvability of Paranthropus Cranial Morphology in Relation to Feeding Biomechanics
Source: Am J Biol Anthropol. 2025 Oct 20;188(2):e70136. doi: 10.1002/ajpa.70136 (PMC12538032; doi:10.1002/ajpa.70136)
Supplement: Supplementary file 1 — Data S1: Supporting Information. [file AJPA-188-e70136-s001.pdf]

Supplementary:

Evaluating the evolvability of *Paranthropus* cranial morphology in relation to feeding biomechanics

Hyunwoo Jung[a,b*], Campbell Rolian[c], David Strait[d,e,f], Karen L. Baab[a]

[a] *Department of Anatomy, College of Graduate Studies, Midwestern University, Glendale, AZ 85308, USA*

[b] *Department of Anthropology, University of California, One Shields Avenue, Davis, CA 95616, USA*

[c] *Department of Anatomy and Cell Biology, McGill University, Montreal, QC H3A 0C7, Canada*

[d] *Department of Anthropology, Washington University in St. Louis, St. Louis, MO 63130, USA*

[e] *Palaeo-Research Institute, University of Johannesburg, Auckland Park, Johannesburg 2092, South Africa*

[f] *DFG Center for Advanced Studies "Words, Bones, Genes, Tools," University of Tübingen, Rümelinstraße 23, D-72070 Tübingen, Germany*

***Corresponding author.**

E-mail address: hwjung@ucdavis.edu (H. Jung).

Supplementary S1. Dietary adaptations in *Paranthropus*.

Analogies to extant mammals exhibiting a more robust feeding apparatus and/or expanded (and flatter) postcanine teeth can also be employed to infer ecomorphology in *Paranthropus* (Andrews et al., 2022:12-13). This ecomorphological approach suggests that the large and robust jaw morphology, as well as low cusped and thickly enameled postcanine teeth seen in robust capuchins (*Sapajus*) compared to gracile capuchin monkeys (*Cebus*) represent biomechanical adaptations for consuming mechanically challenging foods (such as palm nuts in the case of capuchins) (Wright, 2005; Taylor and Vinyard, 2009). In non-primate mammals, analogies also have been made with the giant panda (*Ailuropoda melanoleuca*), known for its diet consisting primarily of bamboo (Du Brul, 1977), a fibrous yet brittle food item (e.g., Xu et al., 2014; Archila et al., 2018). Similarly, comparisons have been drawn with the sea otter (*Enhydra lutra*) as its diet mainly comprises hard foods such as mollusks, crabs, and sea urchins (Walker, 1981; Ziscovici et al., 2014; Constantino et al., 2016; reviewed in Sponheimer et al., 2023). Taken together, ecomorphological inferences indicate that *Paranthropus* underwent biomechanical modification, adapting in particular to challenging or resistant food items.

Yet, there appears to be a conflict between inferred biomechanical adaptations seen in dental morphology and in other indicators of diet (e.g., dental microwear) (e.g., Daegling et al., 2013 vs. Strait et al., 2013). On one hand, the lack of extensive shearing crests on the low-cusped, rounded postcanine occlusal surface in *Paranthropus* does not seem particularly well-adapted for processing tough foods (e.g., Kay, 1977; Lucas, 2004; Ungar 2007; Ungar and Lucas, 2010; Strait et al., 2013; Smith et al., 2015). Rather, such morphology appears better suited for feeding on hard objects (Anthony and Kay, 1993; Meldrum and Kay, 1997; Ungar, 1998; Ungar and Lucas, 2010; Strait et al., 2013; Smith et al., 2015). On the other hand, studies on dental microwear suggest an

absence of hard objects in the diet of at least *P. boisei* (Walker, 1981; Ungar et al., 2008, Grine et al., 2012). In this view, the skull and tooth morphology of *P. boisei* is hypothesized to be an evolutionary compensation for withstanding repetitive muscle loads for processing tough food items, even though it appears suboptimal and contradictory to tooth biomechanics (e.g., Ungar and Hlusko, 2016; Sponheimer et al., 2023). Ungar and Hlusko (2016) argued that increased enamel thickness might evolve more easily (i.e., rapid response to selection) in *P. boisei*, resulting in the absence of shearing crests as an architectural by-product of how enamel is deposited. The hard fallback foods hypothesis (e.g., Scott et al., 2014) during brief periods of resource stress has been proposed to address this issue, but carbon isotope and tooth chipping data suggest that this explanation does not fit well for *P. boisei* (reviewed in Sponheimer et al., 2023). As an additional complication, a debate has ensued regarding the mechanics of microwear formation (Ungar et al., 2008; Grine et al., 2012; Daegling et al., 2013; Lucas et al., 2013; Strait et al., 2013; Xia et al., 2015; Ackermans et al., 2020; Van Casteren et al., 2020), with some researchers suggesting that hard plant tissues (such as those that might have been consumed by *Paranthropus*) are nonetheless too soft to directly produce abrasive wear on enamel surfaces (Lucas et al., 2013; Strait et al., 2013; Van Casteren et al., 2020).

Disagreement persists as to the diets and dietary adaptations of robust australopiths (e.g., Robinson, 1954, 1963; Scott et al., 2005; van der Merwe et al., 2008; Ungar et al., 2008; Cerling et al., 2011; Strait et al., 2013; Daegling et al., 2013; Lucas et al., 2013; Smith et al., 2015; van Casteren et al., 2020; Wynn et al., 2020; Sponheimer et al., 2023). However, regardless of the specific interpretations, there is little dispute that the highly derived skull morphology of *Paranthropus* likely reflected feeding performance and fitness. Interpretations of *Paranthropus* diets in turn have implications for interpretations of their ecological "strategies" (e.g., Vrba, 1980)

namely, whether *Paranthropus* species were stenotopic (ecologically specialized) or eurytopic (ecologically generalized) (Wood and Strait, 2004; Scott et al., 2005). In this context, a specialist is a species with a narrow utilization and tolerance of ecological variables (a dietary specialist would be stenophagic), while a generalist is a species with a broad utilization and tolerance of such variables (euryphagic in relation to food). There is disagreement as to whether or not *Paranthropus* was ecologically generalized or specialized, with some studies pointing to dental microwear, mechanics, and various paleontological variables to suggest that at least some *Paranthropus* species were generalists (e.g., Wood and Strait, 2004; Scott et al., 2005; Smith et al., 2015), while stable carbon isotope studies in particular may indicate dietary specialization (i.e., a substantial reliance of C4 foods) in *P. boisei* (e.g., Cerling et al., 2011). However, there is no doubt that the genus *Paranthropus* as a whole was ecologically generalized with respect to diet (i.e., more variable diet) because *P. robustus* and *P. boisei* differ notably with respect to both their stable carbon isotope and dental microwear signals. Yet, *Paranthropus* species have been viewed as ecological, and particularly dietary, specialists within the hominin clade since the middle of the last century (Robinson, 1954, 1963), an idea that persists in the literature (e.g., Antón et al. 2014).

Degree of ecological flexibility may in turn influence species survivability, because it is thought that although specialists can better exploit their preferred resource than can generalists, generalists are better able to withstand environmental shifts or perturbations, because they are not dependent on a narrow range of resources whose availability might be affected by the ecological change (Vrba, 1980, 1985; Wood and Strait, 2004; Clavel et al., 2011; Raia et al., 2016). From this perspective, the inability to respond to changing selective pressures can be due to morphological, physiological, and behavioral factors (e.g., Schlaepfer et al., 2002). A related idea is that while generalists can have more specialized descendants, specialists rarely give rise to more generalized

species. This is reflected by greater specialization within clades, which may increase their probability of going extinct (Raia et al., 2016). Notably, it has been hypothesized that the extinction of *Paranthropus* is related to it being over-specialized (Wynn et al., 2020).

However, the relationship between the degree of specialization and risk of extinction can be complex (Futuyma and Moreno, 1988) and may depend on factors such as the clade examined and the timescale considered (e.g., Colles et al., 2009; Day et al., 2016). This complexity arises because the relationship between traits, their functions, and evolutionary capacity is inherently non-linear, often making it difficult to determine whether a given trait acts as a constraining specialization or an enabling innovation, since in many cases traits likely serve both roles (Futuyma and Moreno, 1988).

Nevertheless, we are not arguing that specialization or generalization contributed to the extinction of *Paranthropus*, as this question lies beyond the scope of the present study. Furthermore, for our purposes, whether specialists are more or less prone to extinction is irrelevant. Instead, we emphasize a related but distinct concept: that of being "over-derived." This term is intended to describe how *P. boisei*'s highly derived morphology may have placed it in a region of morphospace—or along evolutionary trajectories—from which shifts toward new adaptive peaks were more difficult under certain conditions.

Supplementary S2. The minimum and maximum values of mean trait-wise autonomy (a) and integration.

Within each simulation iteration, the minimum and maximum values of mean trait-wise autonomy (*a*) and integration (variance of eigenvalues; VE) were calculated in addition to their averages. The Spearman's rank correlation coefficients were then computed with the number of generations required to reach the target across 1,000 simulation iterations for each evolutionary scenario. The correlations with the minimum or maximum values were calculated as these extremes may influence the evolutionary rates during certain phases of the simulations involving generation-to-generation selection.

The results showed that the number of generations was negatively correlated with the minimum values of mean *a* and VE in the estimated variance/covariance (V/CV) matrices derived from chimpanzee-like V/CV patterns (Supplementary Table S5). The maximum values of mean *a* and VE showed inconsistent patterns across evolutionary scenarios. With human-like patterns, the number of generations was negatively correlated with both mean *a* and VE, except for the maximum values of VE (Supplementary Table S5). The maximum values of VE generally showed only weak correlations, except in the case of the A.L. 444-2 and OH 5 pair.

From these results, it can be inferred that shorter 'evolutionary times' were generally required to reach the target when the minimum value of mean *a* was higher under both human-like and chimpanzee-like V/CV patterns. Thus, these results may indicate that mean *a* needs to exceed a certain threshold to facilitate evolution, while the degree of constraint on trait evolution at the macroevolutionary scale can vary between human-like and chimpanzee-like patterns.

Furthermore, in most cases, the maximum values of VE in each simulation iteration showed either negligible correlation or low-to-moderate positive correlations (e.g., *P. boisei* – *Au.*

afarensis pair) with the number of generations under both the human-like and chimpanzee-like V/CV patterns. Therefore, although speculative, there may be a hypothetical threshold or a non-linear relationship beyond which excessively strong integration may further constrain populations under generation-to-generation selection—possibly by reducing the flexibility of simulated populations to achieve better alignment in subsequent generations.

Taken together, the results in the current simulation study may suggest that integration that is sufficiently strong without being excessive, and trait autonomy that surpasses a certain threshold facilitate evolution. This interpretation is consistent with the theoretical perspective that moderate levels of integration best promote evolvability (Hansen, 2003; reviewed in Jablonski, 2022). However, caution is warranted as the current simulation study does not demonstrate the direct influence of the evolutionary properties of the V/CV matrix (e.g., extreme VE values) on populations in immediate subsequent generations, making the above interpretation speculative and limited. Further studies are needed to investigate the direct effects of (extreme) levels of integration on microevolutionary evolvability (Hansen and Houle, 2008) and evolutionary flexibility (Marroig et al., 2009) in populations across both immediate and more distant subsequent generations, and ultimately on macroevolutionary evolvability.

Supplementary Table S1. Landmark definitions.

Landmark	Definition
Midzygomatic arch outer (ZAO)	Point on outer surface of arch halfway along free part of arch (bilateral landmarks)
Midzygomatic arch inner (ZAI)	Point on inner surface of arch halfway along free part of arch directly medial to ZAO
Midzygomatic arch superior (ZAS)	point on superior border of arch halfway along free part of arch (same plane as ZAO/ZAI)
Midzygomatic arch inferior (ZAI)	point on inferior border of arch halfway along free part of arch (same plane as ZAO/ZAI)
Posterior temporal foramen (PTF)	Posteriormost point of temporal foramen on anterior margin of supraglenoid gutter
Anterior temporal foramen (ATF)	Anteriormost point of temporal foramen on zygomatic bone on same plane as PTF, ZAO, and ZAI
Lateral temporal foramen (LTF)	Taken on inner surface of zygomatic arch at position of maximal breadth of temporal foramen at same plane as PTF and ATF
Medial temporal foramen (MTF)	Taken on infratemporal crest (junction of sphenoid squama and infratemporal surface of sphenoid) at same coronal plane as LTF
Posterior temporalis (PT)	Posteriormost point on temporalis origin (superior temporal line) in Frankfort Horizontal (FH), at edge of crest if present
Anterior temporalis (AT)	Anteriormost point on temporalis origin (superior temporal line) in FH, may extend onto the posterior border of the browridge
Superior temporalis (ST)	Identify where the temporalis muscle is tallest over PTF in a plane perpendicular to FH (this is the point at the top of that line on the superior temporal line)
Inferior temporalis (IT)	Point on zygomatic arch directly inferior to ST when oriented in FH
Articular eminence (AE)	Apex (inferiormost point) of mid-articular eminence when oriented in FH along same parasagittal plane as PG (articular eminence is in front of mandibular fossa and projects down as a bar of bone)
Masseteric tubercle (MT)	Anteriormost point of rugosity of masseter attachment on inferior surface of zygomatic arch or zygomatic bone
Orbitale (O)	Lowest point of orbit in frontal view
Mid-zygomaticoalveolar crest (ZAC)	Midpoint of zygomaticoalveolar crest (bone from alveolar margin to zygomatic arch) in frontal view
Frontomalaretemporale (FMT)	Lateralmost point on frontomalare (or frontozygomatic) suture (take along sharp edge of temporal crest—at the junction of the side of the orbit and the back of the orbit)
Jugale (J)	Vertex of angle between frontal and temporal processes of zygomatic bone

Infero-lateral orbit (IO)	Inferolateral 'corner' of orbit (imagine the orbit is a square and take the point at the angle on the bottom and to the side furthest from the midline)
Anterior zygomaxillary (AZM)	Anterior point on 'cheek' in midline plane through ATF at maximal thickness perpendicular to long axis of section (taken inferiorly, below orbit)
Posterior root (PR)	Point on posterior surface of the root of the zygomatic arch in same horizontal plane as FH
Buccal P^4 (BMP4)	Buccalmost point on P^4 crown
Lingual P^4 (LMP4)	Lingualmost point on P^4 crown
Buccal M^2 (BMM2)	Buccalmost point on M^2 crown
Lingual M^2 (LMM2)	Lingualmost point on M^2 crown
Maxillary C^1/P^3 alveolar margin (C-P3)	Buccal alveolar margin between C^1 and P^3
P^4/M^1 alveolar margin (P4-M1)	Buccal alveolar margin between P^4 and M^1
Distal M^3 alveolar margin (DM3)	Buccal alveolar margin at level of distal M^3
Mesial C^1 alveolar margin (MC)	Buccal alveolar margin at level of mesial C^1 (I^2/C^1 contact except in case of canine diastema)
Distal I^2 alveolar margin (DI2)	Buccal alveolar margin at level of distal I^2 (lateral incisor; this maybe equivalent to MC if there is no diastema; bilateral landmarks)
Postglenoid process (PG)	Tip of postglenoid process (take in center if process is flat; postglenoid process is behind mandibular fossa and projects down)
Mandibular fossa (MF)	Deepest point (nadir) of mandibular fossa in center of fossa when oriented in FH along same parasagittal plane as PG
Ectomolare2 (EM2)	Buccal alveolar margin centered above M^2
Ectopremolare4 (EP4)	Buccal alveolar margin centered above P^4
Prosthion (P)	Buccal alveolar margin between I^1's
Entopremolare3 (NP3)	Lingual alveolar margin at mid-P^3 (bilateral landmarks)
Entomolare2 (NM2)	Lingual alveolar margin at mid-M^2 (bilateral landmarks)

Supplementary Table S2. Biomechanical measurements in the cranium.

#	Measurement	Definition	Mechanical significance	Landmark in Supplementary Table S1
1	Breadth of midzygomatic arch (Br_midzygo)	Midzygomatic arch outer to midzygomtic arch inner	Bending strength in transverse plane (i.e., resisting medial component of deep masseter force vector)	ZAO:ZAI
2	Height of midzygomatic arch (Ht_midzygo)	Midzygomatic arch superior to midzygomatic arch inferior, as measured between the superior-most and inferior-most points on the zygomatic arch in the same coronal plane as midzygomatic arch outer	Bending strength in sagittal plane (i.e., resisting vertical components of superficial and deep masseter force vectors)	ZAS:ZAI
3	Maximum length of temporal foramen (MaxLn_tempfora)	Posterior-most point on the anterior margin of the supraglenoid gutter (posterior temporal foramen) to the anterior-most point of the temporal foramen on the zygomatic bone (anterior temporal foramen) in the same plane as ZAO and ZAI	Related to temporal foramen size; roughly proportional to temporalis cross sectional area, should be correlated with maximum force of temporalis	PTF:ATF
4	Maximum breadth of the temporal foramen (MaxBr_tempfora)	Maximum breadth of the temporal foramen from the inner surface of the zygomatic arch (lateral temporal foramen) to the neurocranial wall in the same coronal plane (medial temporal foramen)	Related to temporal foramen size; roughly proportional to temporalis cross sectional area, should be correlated with maximum force of temporalis	LTF:MTF

5	Bi-zygomatic breadth (Bi_zygo)	Right to left midzygomatic arch outer	Related to size of temporalis and orientation of deep masseter, as well as the bending moment of the zygomatic arch in the coronal plane	ZAO_left:ZAO_right
6	Maximum length of temporalis origin (MaxLn_tempmuscle)	Posterior-most point on temporalis origin (posterior temporalis) to anterior-most point on temporalis origin (anterior temporalis)	Related to shape of temporalis origin; approximates relative sizes of anterior vs. posterior portions of temporalis muscle	PT:AT
7	Perpendicular height of temporalis origin (Ht_tempmuscle)	Maximum height of the temporalis origin perpendicular to its chord of maximum length, from the superior temporal line (superior temporalis) to the zygomatic arch (inferior temporalis)	Related to shape of temporalis origin; approximates relative sizes of anterior vs. posterior portions of temporalis muscle	ST:IT
8	Articular eminence to masseteric tubercle (AE_MT)	Apex of mid-articular eminence to masseteric tubercle	Roughly proportional to superficial masseter lever arm, as well as orientation of superficial masseter (as length increases proportionally, superficial masseter may become more inclined); inversely proportional to gape	AE:MT
9	Zygomaticoalveolar expansion (Zygomatico_exp)	Distance from orbitale to mid-zygomaticoalveolar crest in frontal view	Resistance to shear in coronal plane due to contraction of masseter	O:ZAC
10	Frontomalaretemporale to vertex zygomatic angle (FMT_J)	Frontomalaretemporale to jugale	Related to zygomatic angle; resistance to bending stress at the angle due to contraction of masseter because an oblique angle implies the existence of an expansion of bone at the vertex	FMT:J

11	Vertex zygomatic angle to superior point of mid-zygomatic arch (J_ZAS)	Jugale to midzygomatic arch superior	Related to zygomatic angle; resistance to bending stress due to contraction of masseter because an oblique angle implies the existence of an expansion of bone at the vertex	J:ZAS
12	Frontomalaretemporale to superior point of mid-zygomatic arch (FMT_ZAS)	Frontomalaretemporale to midzygomatic arch superior	Related to zygomatic angle; resistance to bending stress due to contraction of masseter because an oblique angle implies the existence of an expansion of bone at the vertex	FMT:ZAS
13	Inferolateral breadth of postorbital bar (PO_Br)	From jugale to infero-lateral 'corner' of orbit	Related to zygomatic angle; resistance to bending stress due to contraction of masseter	Z:IO
14	Minimum thickness of zygomatic root (AZM_PR)	Minimum distance between a point on 'cheek' in midline plane through anterior temporal foramen at maximal thickness perpendicular to long axis of section and a point on the posterior surface of the root of the zygomatic arch in same horizontal plane as Frankfort Horizontal	Resistance to bending in transverse plane as superficial masseter contracts	AZM:PR
15	Maximum maxillary P^4 breadth (P4_Br)	Maximum buccolingual chord distance between buccal-most and lingual-most points on P^4 crown	Related to tooth crown strength (resistance to ribbon fractures), rate of wear, and occlusal area	BMP4: LMP4
16	Maximum maxillary M^2 breadth (M2_Br)	Maximum buccolingual chord distance between buccal-most and lingual-most points on M^2 crown	Related to tooth crown strength (resistance to ribbon fractures), rate of wear, and occlusal area	BMM2:LMM2
17	Maxillary premolar alveolar length (P4_alveolar_Ln)	C^1-P^3 buccal alveolar crest to P^4-M^1 buccal alveolar crest	Surrogate for mesiodistal length of premolar crowns, related to tooth crown strength (resistance to ribbon fractures), rate of wear, and occlusal area	C-P3:P4-M1

18	Maxillary molar alveolar length (Molar_alveolar_Ln)	P^4-M^1 buccal alveolar crest to distal M^3 buccal alveolar crest	Surrogate for mesiodistal length of molar crowns, related to tooth crown strength (resistance to ribbon fractures), rate of wear, and occlusal area	P4-M1:DM3
19	Maxillary canine alveolar length (Canine_Ln)	C^1-P^3 buccal alveolar crest to I^2-C^1 buccal alveolar crest (or mesial C^1 buccal alveolar crest if diastema is present)	Correlate of social signaling, and possibly gape insofar as canine crown base dimensions may be correlated with canine projection	C-P3:MC
20	Maxillary bilateral incisor alveolar length (Bilat_Incisor)	Breadth between right and left C^1/I^2 buccal alveolar crests (or distal I^2 buccal alveolar crests if diastema is present)	Functional surface for incision	DI2_left:DI2_right
21	Postglenoid process to mandibular fossa (PG_MF)	Tip of postglenoid process to nadir of mandibular fossa in the same parasagittal plane	Related to articular eminence angle; related to depth of the fossa and inclination of the articular eminence, which should be correlated with the orientation of the joint reaction force at the temporomandibular joint (TMJ), which in turn should be related to the orientation of the superficial masseter muscle	PG:MF
22	Mandibular fossa to articular eminence (MF_AE)	Nadir of mandibular fossa to apex of articular eminence in the same parasagittal plane as tip of postglenoid process	Related to articular eminence angle; related to depth of the fossa and inclination of the articular eminence, which should be correlated with the orientation of the joint reaction force at the temporomandibular joint (TMJ), which in turn should be related to the orientation of the superficial masseter muscle	MF:AE
23	Postglenoid process to articular eminence (PG_AE)	Tip of postglenoid process to apex of articular eminence in the same parasagittal plane	Related to articular eminence angle; related to depth of the fossa and inclination of the articular eminence, which should be correlated with the orientation of the joint reaction force at the temporomandibular joint	PG:AE

			(TMJ), which in turn should be related to the orientation of the superficial masseter muscle	
24	Articular eminence to ectomolare2 (AE_EM2)	Apex of articular eminence to the buccal alveolar margin centered above the maxillary M^2 (ectomolare2)	Approximates the M^2 load arm	AE:EM2
25	Articular eminence to ectopremolare4 (AE_EP4)	Apex of articular eminence to the buccal alveolar margin centered above the maxillary P^4 (ectopremolare4)	Approximates the P^4 load arm	AE:EP4
26	Articular eminence to prosthion (AE_P)	Apex of articular eminence to the buccal alveolar margin centered between the maxillary I^1s (prosthion)	Approximates the I^1 load arm	AE:P
27	Mechanical palate protrusion (Palate_Protrusion)	Articular eminence to prosthion (26) minus articular eminence to mid-zygomaticoalveolar crest	Moment arm of rostrum when bent/sheared in sagittal plane at incisors	AE:P - AE:ZAC
28	Inner palate breadth at P^3 (Br_palate_P3)	Breadth between lingual alveolar margins at mid-P^3 (entopremolare3)	Resistance to torsion during premolar bites	NP3_right:NP3_left
29	Inner palate breadth at maxillary M^2 (Br_Palate_M2)	Breadth between lingual alveolar margins at mid-M^2 (entomolare2)	Related to the Constrained Lever Model of jaw biomechanics (narrow tooth rows allow higher maximum bite force in bites in Zone II [i.e., bites on the molars])	NM2_right:NM2_left

Supplementary Table S3. Correlation coefficient between p_{max} (the first principal component axis; PC 1) and vector of the evolutionary change (Δz) between fossil hominins. We measured the correlation coefficient, calculated as the cosine of the angle (θ) between the evolutionary gradient (vector of the multivariate difference in means) between pairs of extinct hominin species (Δz) and the normalized PC 1 eigenvector for each extant species (direction of greatest phenotypic variance; p_{max}) (Schluter, 1996). The absolute value of the coefficient was used to quantify the magnitude of correlation, not its direction. This coefficient assesses how closely species divergence is aligned with the primary axis of variation within a species. A coefficient near zero indicates poor alignment of the vectors, whereas a value closer to one means that they are closely aligned and p_{max} may facilitate rapid change between species.[%]

	p_{max} of extant taxon	
Evolutionary scenario[#]	*Homo sapiens*	*Pan troglodytes*
Pb and Aa	0.32 (0.17-0.47)[*]	0.42 (0.29-0.52)
Pb and Hh	**0.84 (0.67-0.91)**	**0.93 (0.88-0.94)**
Aa and Hh	**0.83 (0.66-0.93)**	**0.94 (0.87-0.95)**

[*]Observed correlation coefficient and the 2.5–97.5th percentile range from 1,000 bootstrapping procedures in parenthesis. Observed values that fall outside the 2.5–97.5th percentile range of the comparison taxon are shown in bold.

[#]Abbreviations: Pb = *Paranthropus boisei* (OH 5); Aa = *Australopithecus afarensis* (A.L. 444-2); Hh = *Homo habilis* (KNM-ER 1813).

[%]The correlation coefficient between the *H. sapiens* p_{max} and Δz calculated for all pairs of fossil hominins was significantly smaller ($p<0.05$) than the same values calculated with the *P. troglodytes* p_{max}, except for the OH 5 and A.L. 444-2 pair. Furthermore, the correlation coefficients between p_{max} (based on humans or chimpanzees) and the OH 5 and A.L. 444-2 Δz are smaller than comparisons using other fossil hominin pairs, which is consistent with the observation that OH 5 and A.L. 444-2 are roughly aligned with the axes of within-species variation in the PC

ordinations. The results of *c* (Table 3) in this study may suggest the need to consider the entire trait covariance or correlation structure, rather than focusing solely on p_{max} (Hansen and Houle, 2008). If we relied solely on the comparisons of p_{max} and Δz, we might have concluded that evolution would be faster using the chimpanzee V/CV matrix, which was not the case. Measures such as *e* and *c* better account for the covariance structure of the extant proxies and explain the larger number of generations based on the *P. troglodytes* V/CV matrix in the simulations.

Supplementary Table S4. The original principal component (PC) coefficients for the first three axes of the extant taxa and the two fossil hominins used in the simulations. Abbreviations as in Supplementary Table S2.

#	Traits	Fossil hominins included in the simulations								
		H. habilis – P. boisei			*Au. afarensis – P. boisei*			*Au. afarensis – H. habilis*		
		PC 1 (65%)	PC 2 (15%)	PC 3 (3%)	PC 1 (67%)	PC 2 (19%)	PC 3 (4%)	PC 1 (66%)	PC 2 (17%)	PC 3 (4%)
1	Br_midzygo	-0.012	0.030	0.056	-0.009	0.039	-0.046	-0.012	0.025	-0.029
2	Ht_midzygo	-0.015	0.029	-0.036	-0.009	0.048	0.011	-0.010	0.051	0.026
3	MaxLn_tempfora	0.199	0.111	0.037	0.204	0.098	-0.044	0.202	0.113	-0.070
4	MaxBr_tempfora	0.136	0.117	0.107	0.139	0.099	0.015	0.133	0.081	0.059
5	Bi_zygo	0.055	0.428	0.480	0.079	0.444	-0.250	0.061	0.395	-0.138
6	MaxLn_tempmuscle	-0.426	**0.630**	-0.227	-0.393	**0.652**	0.115	-0.409	**0.675**	-0.026
7	Ht_tempmuscle	0.019	0.494	**-0.405**	0.034	0.432	**0.558**	0.024	0.456	**0.564**
8	AE_MT	0.153	0.163	0.367	0.167	0.193	-0.374	0.157	0.157	-0.373
9	Zygomatico_exp	0.226	0.064	-0.014	0.230	0.058	0.075	0.224	0.029	0.124
10	FMT_J	0.044	0.057	0.061	0.045	0.045	0.035	0.044	0.048	0.020
11	J_ZAS	0.159	0.011	0.108	0.160	0.013	-0.079	0.158	0.002	-0.086
12	FMT_ZAS	0.022	0.110	0.104	0.025	0.101	-0.027	0.023	0.103	-0.039
13	PO_Br	0.012	0.119	0.019	0.019	0.120	0.007	0.015	0.119	0.029
14	AZM_PR	-0.048	0.056	0.076	-0.045	0.059	-0.064	-0.049	0.041	-0.045
15	P4_Br	0.015	0.026	0.027	0.017	0.031	-0.016	0.015	0.021	-0.009
16	M2_Br	0.010	0.033	0.039	0.012	0.038	-0.023	0.009	0.022	-0.009
17	P4_alveolar_Ln	0.030	0.034	0.012	0.033	0.038	0.005	0.029	0.016	0.020
18	Molar_alveolar_Ln	0.039	0.069	0.119	0.045	0.087	-0.103	0.038	0.047	-0.090
19	Canine_Ln	0.141	-0.004	-0.137	0.137	-0.033	0.165	0.139	-0.015	0.158
20	Bilat_Incisor	0.221	-0.020	-0.215	0.217	-0.045	0.218	0.219	-0.031	0.205
21	PG_MF	-0.029	0.036	0.042	-0.028	0.037	-0.033	-0.030	0.031	-0.028
22	MF_AE	0.030	0.040	-0.062	0.032	0.033	0.101	0.030	0.026	0.127
23	PG_AE	0.054	0.052	-0.074	0.053	0.028	0.132	0.051	0.025	0.158
24	AE_EM2	0.280	0.111	0.144	0.292	0.139	-0.247	0.291	0.168	-0.307
25	AE_EP4	0.268	0.157	0.199	0.282	0.184	-0.293	0.277	0.192	-0.344
26	AE_P	**0.534**[a]	0.158	-0.129	**0.540**	0.121	0.062	**0.538**	0.155	0.004
27	Palate_Protrusion	0.311	0.016	-0.397	0.306	-0.039	0.344	0.312	0.018	0.289
28	Br_palate_P3	0.193	-0.019	-0.199	0.186	-0.061	0.259	0.190	-0.037	0.267
29	Br_Palate_M2	-0.083	0.094	0.043	-0.082	0.076	0.025	-0.084	0.076	0.034

[a] Trait with highest PC coefficient in each PC axis is bolded.

Supplementary Table S5. The Spearman's rank correlation coefficients between mean trait-wise autonomy (*a*) or integration (variance of eigenvalues; VE) of evolving populations and the number of generations required to reach the target in the simulations. The minimum (Min) and maximum (Max) values of mean *a* and VE, as well as the number of generations, were calculated from each simulation iteration (i.e., 1,000 values for each statistic per evolutionary scenario).

	Estimated variance/covariance (V/CV) matrix							
	Human-like V/CV patterns				Human-like V/CV patterns			
Evolutionary	Mean *a*		VE		Mean *a*		VE	
scenario[#]	Min	Max	Min	Max	Min	Max	Min	Max
Aa to Pb	-0.48	-0.53	-0.14	0.28	-0.32	-0.18	-0.45	0.22
Pb to Aa	-0.43	-0.52	-0.32	0.26	-0.16	-0.01	-0.65	-0.02
Hh to Pb	-0.38	-0.49	-0.17	-0.05	-0.06	-0.01	-0.62	-0.10
Pb to Hh	-0.32	-0.29	-0.11	0.09	-0.19	0.23	-0.54	0.08
Hh to Aa	-0.39	-0.29	-0.24	-0.01	-0.01	0.36	-0.71	-0.21
Aa to Hh	-0.21	-0.02	-0.01	-0.02	-0.21	0.23	-0.37	0.12

[#]Abbreviations: Pb = *Paranthropus boisei* (OH 5); Aa = *Australopithecus afarensis* (A.L. 444-2); Hh = *Homo habilis* (KNM-ER 1813).

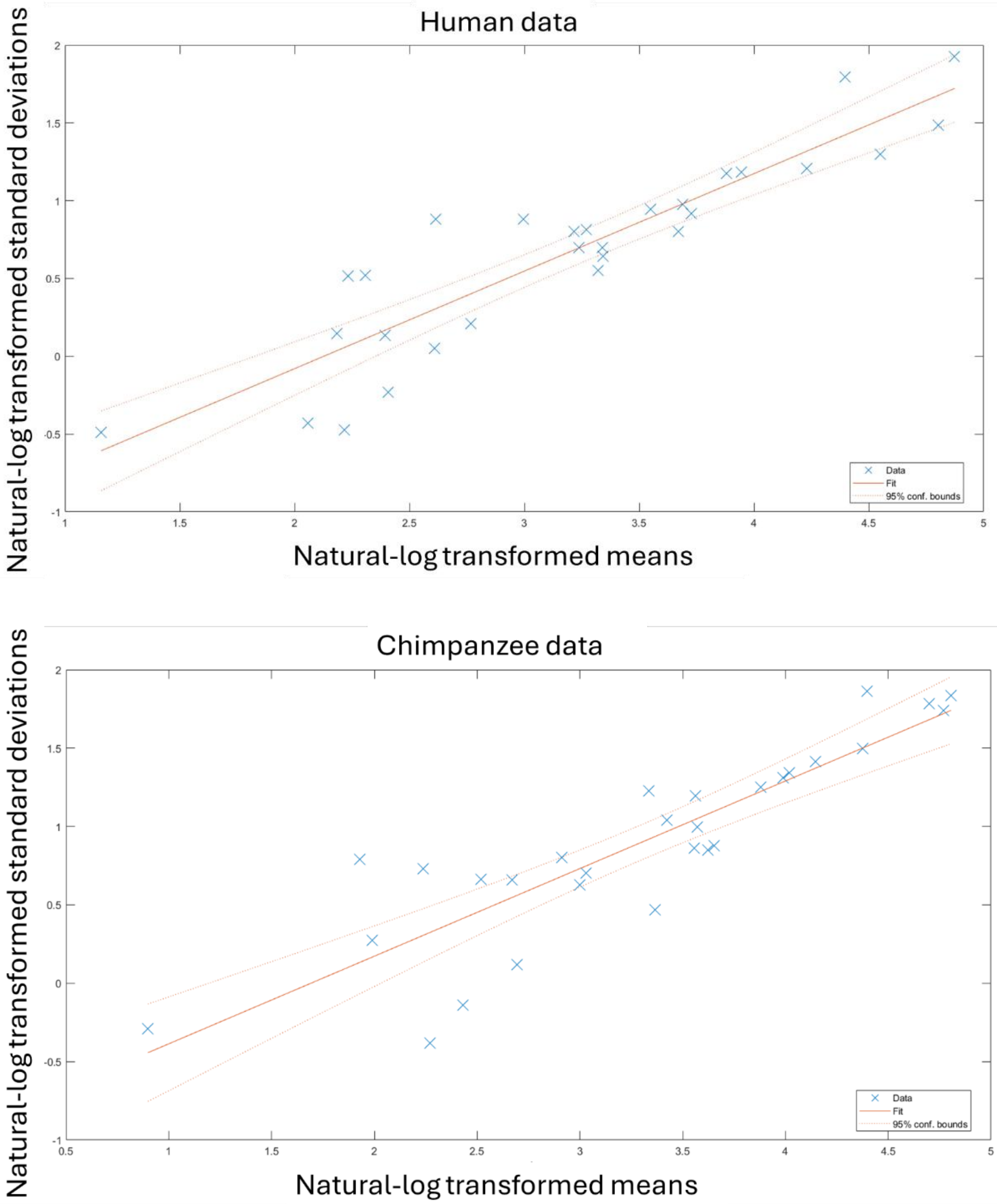

Supplementary Figure S1. Linear relationship between the natural-log transformed trait mean and its standard deviation of humans (adjust-R square=0.82) and chimpanzees (adjust-R square=0.76). Each plot displays 29 data points, represented by X symbols (one for each trait per taxon). The red solid line indicates the regression fit, while the red dotted lines represent the 95% confidence bounds.

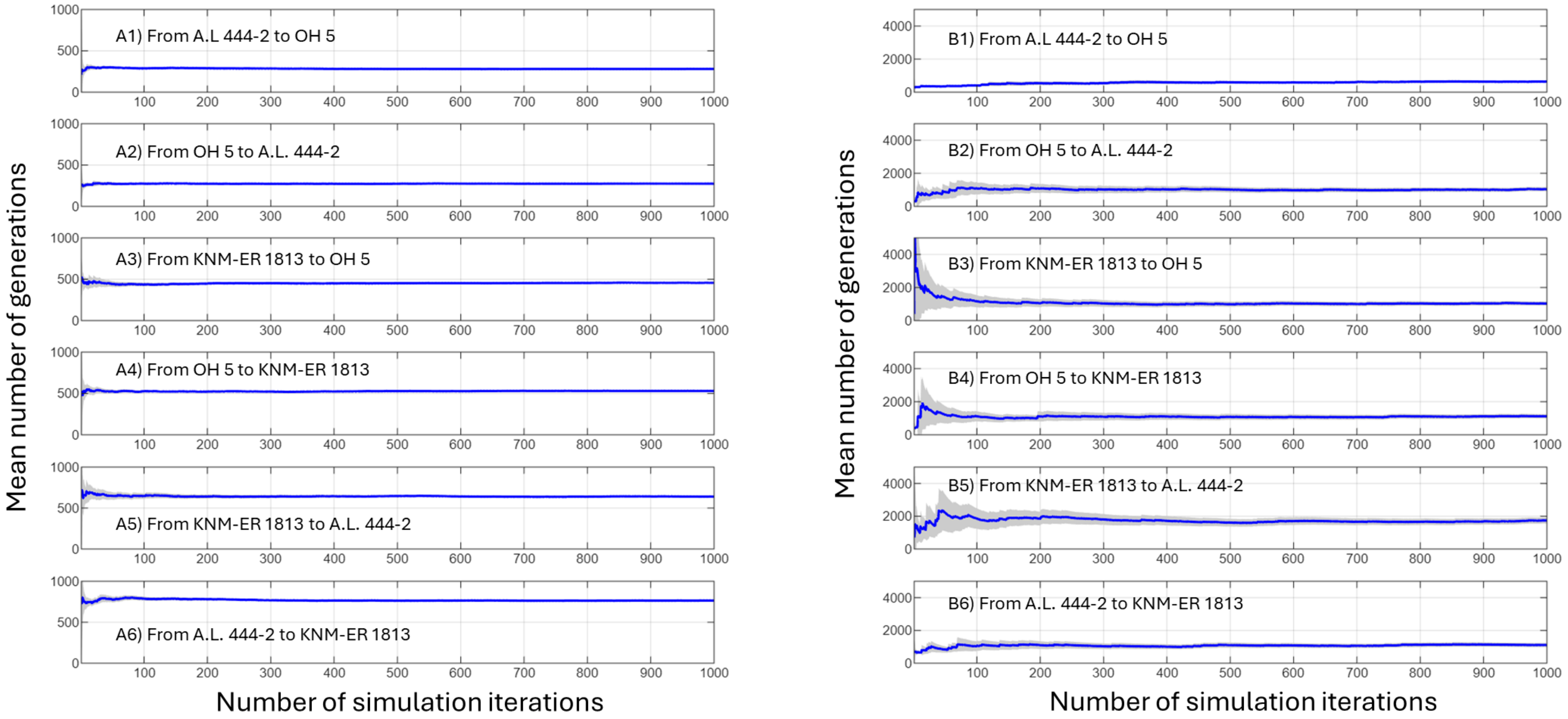

Supplementary Figure S2. Cumulative means of number of generations. Blue lines represent the cumulative means of number of generations, and the gray shading indicates the 95% confidence intervals calculated from the estimated variance/covariance (V/CV) matrix derived from trait V/CV pattern in A) human and B) chimpanzee.

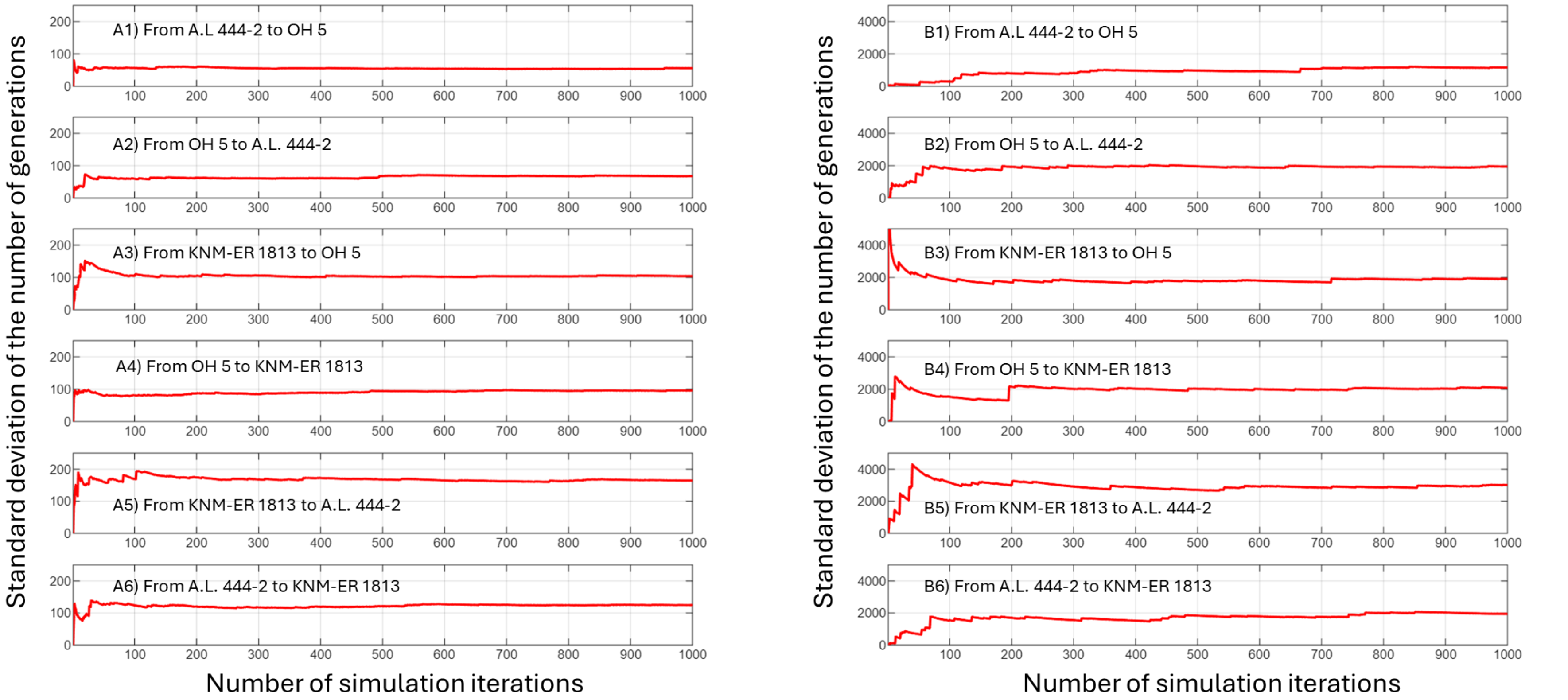

Supplementary Figure S3. Cumulative standard deviations (SDs) of the number of generations. Red lines represent the cumulative SD of number of generations calculated from the estimated variance/covariance (V/CV) matrix derived from the trait V/CV pattern in (A) humans and (B) chimpanzees.

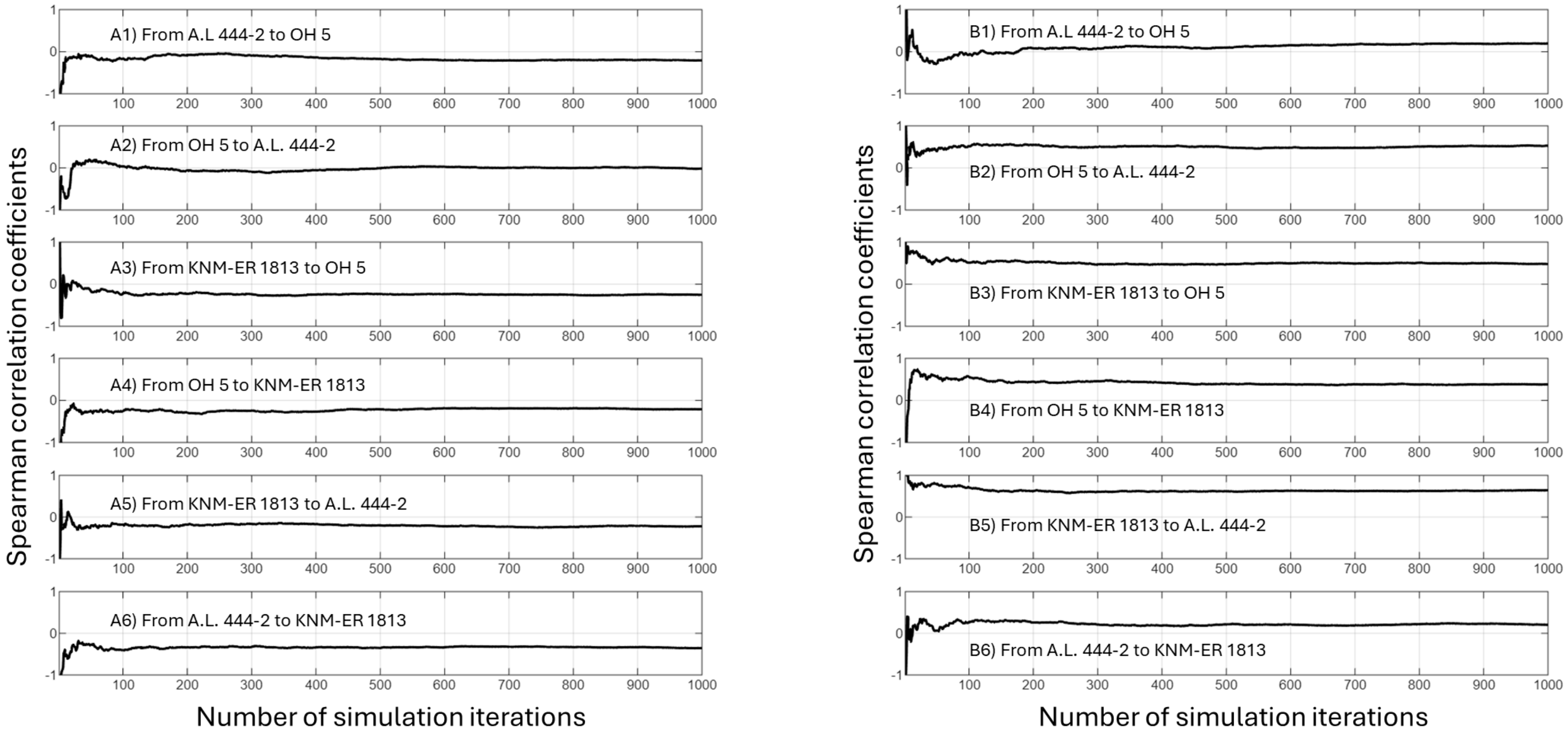

Supplementary Figure S4. Cumulative Spearman correlation coefficients between the average values of mean trait-wise autonomy (*a*) and the number of generations. Black lines represent the cumulative correlation coefficients calculated from the estimated variance/covariance (V/CV) matrix derived from the trait V/CV pattern in (A) humans and (B) chimpanzees.

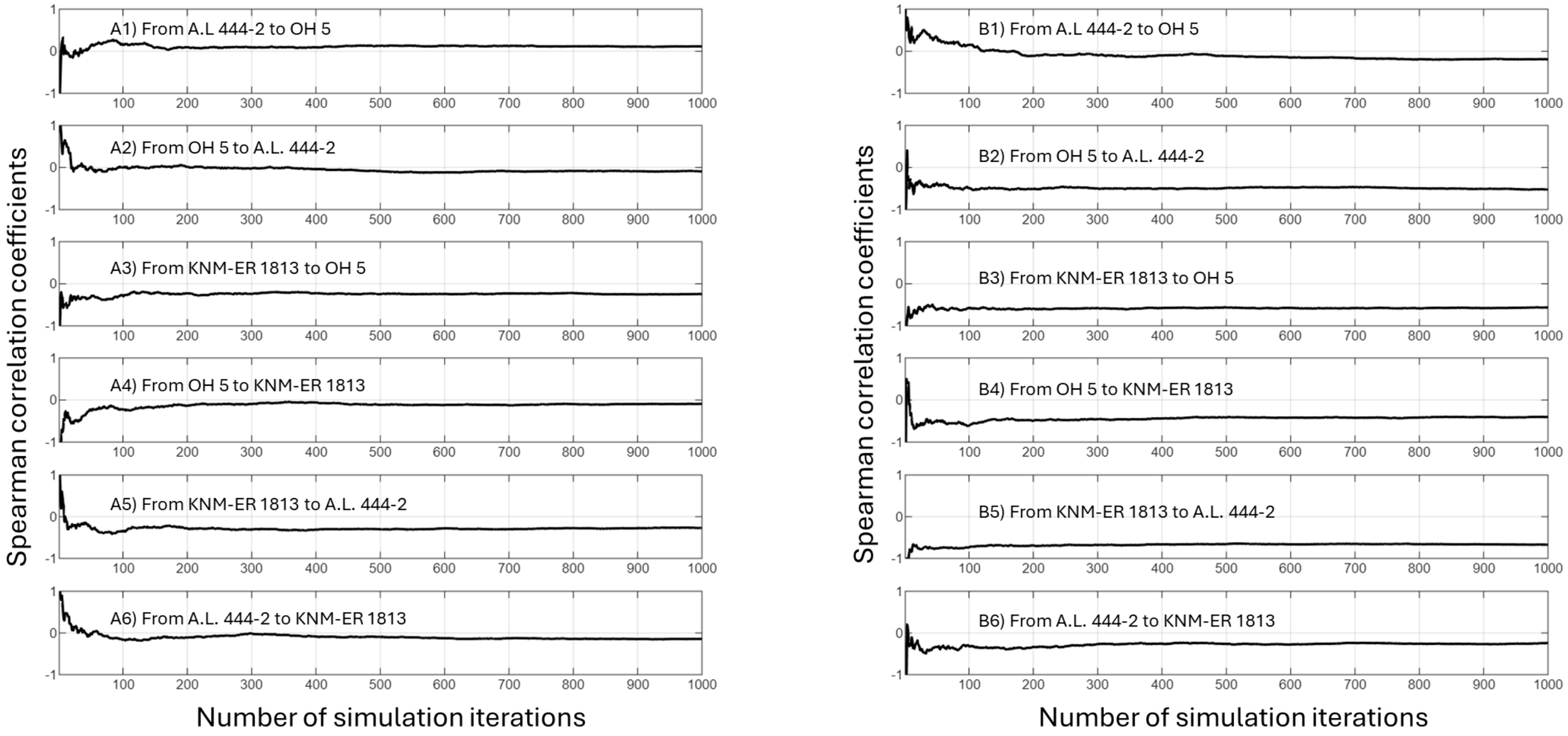

Supplementary Figure S5. Cumulative Spearman correlation coefficients between the average values of integration (variance of eigenvalues; VE) and the number of generations. Black lines represent the cumulative correlation coefficients calculated from the estimated variance/covariance (V/CV) matrix derived from the trait V/CV pattern in (A) humans and (B) chimpanzees.

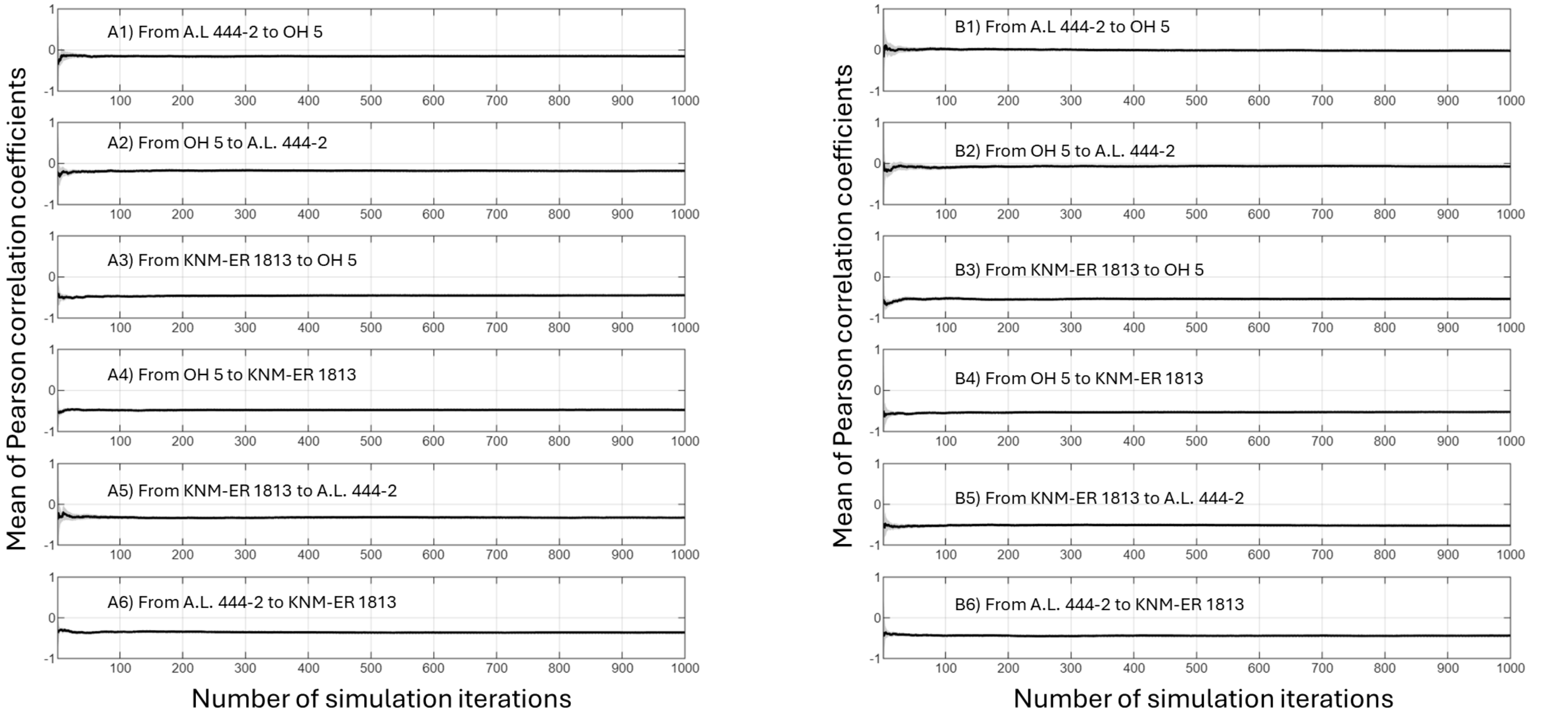

Supplementary Figure S6. Cumulative means of Pearson correlation coefficients between mean trait-wise autonomy (*a*) and the degree of morphological change. Pearson correlation coefficient within each simulation iteration was calculated on a scale from 0% (start of the simulation) to 100% (end of the simulation). Black lines represent the cumulative means of correlation coefficients, and the gray shading indicates the 95% confidence intervals calculated from the estimated variance/covariance (V/CV) matrix derived from the trait V/CV pattern in (A) humans and (B) chimpanzees.

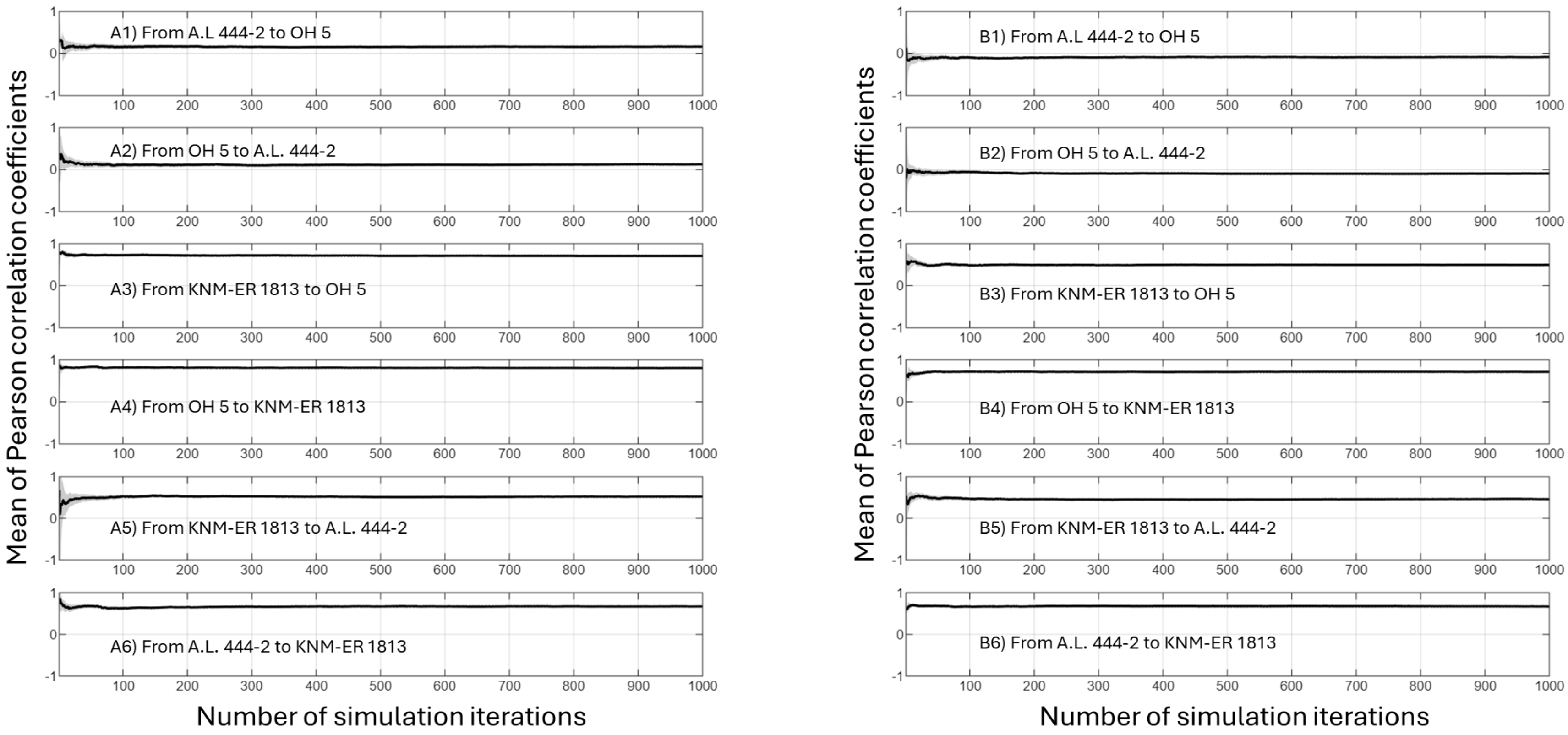

Supplementary Figure S7. Cumulative means of Pearson correlation coefficients between integration (variance of eigenvalues; VE) and the degree of morphological change. Pearson correlation coefficient within each simulation iteration was calculated on a scale from 0% (start of the simulation) to 100% (end of the simulation). Black lines represent the cumulative means of correlation coefficients, and the gray shading indicates the 95% confidence intervals calculated from the estimated variance/covariance (V/CV) matrix derived from the trait V/CV pattern in (A) humans and (B) chimpanzees.

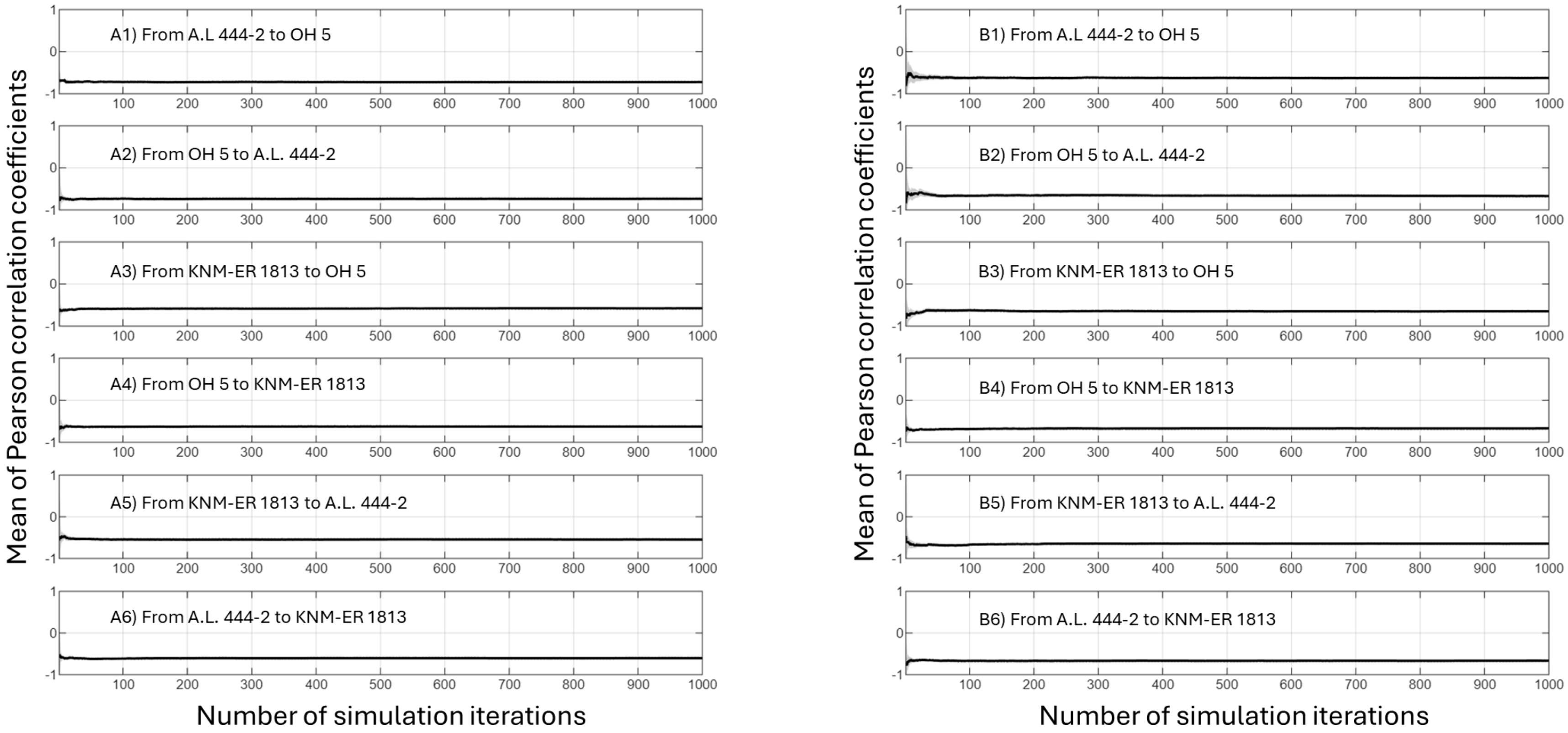

Supplementary Figure S8. Cumulative means of Pearson correlation coefficients between mean trait-wise autonomy (*a*) and integration (variance of eigenvalues; VE). Pearson correlation coefficient within each simulation iteration was calculated on a scale from 0% (start of the simulation) to 100% (end of the simulation). Black lines represent the cumulative means of correlation coefficients, and the gray shading indicates the 95% confidence intervals calculated from the estimated variance/covariance (V/CV) matrix derived from the trait V/CV pattern in (A) humans and (B) chimpanzees.

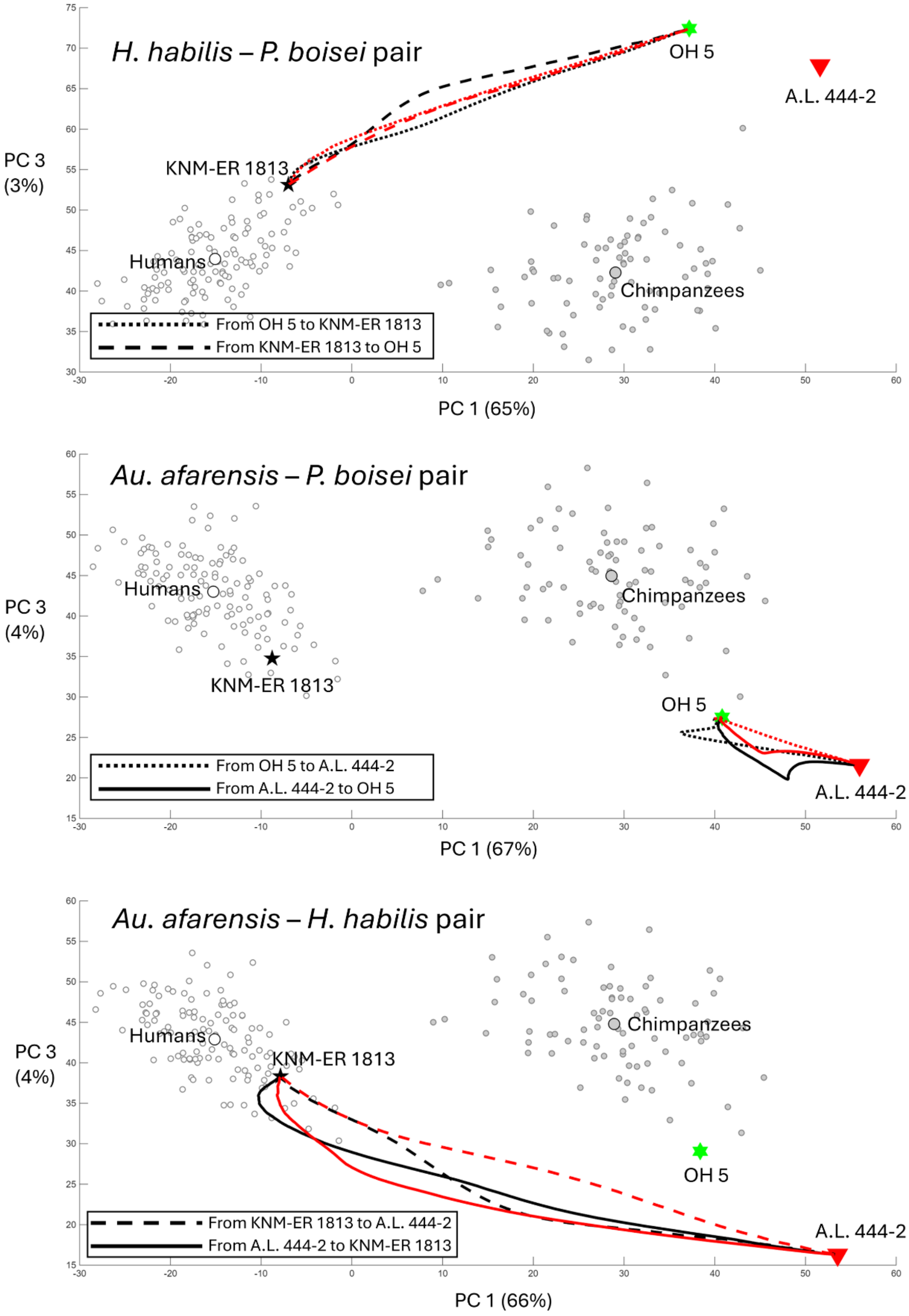

Supplementary Figure S9. Scatter plots of the principal component (PC) 1 and 3 of extant taxa and fossil hominins, along with the average evolutionary trajectories from the simulations. PC

plots are generated using data from *Homo sapiens*, *Pan troglodytes*, and two fossil hominins included in the simulations. The fossil hominin not included in the simulations is projected into the PC morphospace. White and gray circles present *Homo sapiens* and *Pan troglodytes* specimens, respectively, in PC plots with the centroid with larger size and less opacity. Black pentagram: KNM-ER 1813 (*Homo habilis*); Green hexagram: OH 5 (*Paranthropus boisei*); Red triangle: A.L. 444-2 (*Australopithecus afarensis*); Red lines: evolutionary trajectories based on the estimated variance/covariance (V/CV) matrix using human trait V/CV pattern; Black lines: evolutionary trajectories based on the estimated V/CV matrix using chimpanzee trait V/CV pattern; Solid lines: trajectories from *Au. afarensis* to other hominins; Dotted lines: trajectories from *P. boisei* to other hominins; Dashed lines: trajectories from *H. habilis* to other hominins.

Supplementary references

Ackermans, N. L., Winkler, D. E., Martin, L. F., Kaiser, T. M., Clauss, M., & Hatt, J.-M. (2020). Dust and grit matter: abrasives of different size lead to opposing dental microwear textures in experimentally fed sheep (*Ovis aries*). *Journal of Experimental Biology, 223*(3), jeb220442.

Andrews, P., Reynolds, S. C., & Bobe, R. (2022). Approaches to the study of past environments. In S. C. Reynolds & R. Bobe (Eds.), *African Paleoecology and Human Evolution* (pp. 7-14). New York: Cambridge University Press.

Anthony, M. R., & Kay, R. F. (1993). Tooth form and diet in ateline and alouattine primates; reflections on the comparative method. *American Journal of Science*, *293*(A), 356-382.

Antón, S. C., Potts, R., & Aiello, L. C. (2014). Evolution of early *Homo*: An integrated biological perspective. *Science, 345*(6192), 1236826. doi:10.1126/science.1236828

Archila, H., Kaminski, S., Trujillo, D., Zea Escamilla, E., & Harries, K. A. (2018). Bamboo reinforced concrete: a critical review. *Materials and Structures, 51*, 1-18.

Cerling, T. E., Mbua, E., Kirera, F. M., Manthi, F. K., Grine, F. E., Leakey, M. G., . . . Uno, K. T. (2011). Diet of *Paranthropus boisei* in the early Pleistocene of East Africa. *Proceedings of the National Academy of Sciences, 108*(23), 9337-9341.

Cheverud, J. M. (1988). A comparison of genetic and phenotypic correlations. *Evolution*, *42*(5), 958-968.

Clavel, J., Julliard, R., & Devictor, V. (2011). Worldwide decline of specialist species: toward a global functional homogenization?. *Frontiers in Ecology and the Environment*, *9*(4), 222-228.

Colles, A., Liow, L. H., & Prinzing, A. (2009). Are specialists at risk under environmental change? Neoecological, paleoecological and phylogenetic approaches. *Ecology Letters*, *12*(8), 849-863.

Constantino, P. J., Bush, M. B., Barani, A., & Lawn, B. R. (2016). On the evolutionary advantage of multi-cusped teeth. *Journal of the Royal Society Interface, 13*(121), 20160374.

Daegling, D. J., Judex, S., Ozcivici, E., Ravosa, M. J., Taylor, A. B., Grine, F. E., . . . Ungar, P. S. (2013). Viewpoints: feeding mechanics, diet, and dietary adaptations in early hominins. *American Journal of Physical Anthropology, 151*(3), 356-371. doi:10.1002/ajpa.22281

Day, E. H., Hua, X., & Bromham, L. (2016). Is specialization an evolutionary dead end? Testing for differences in speciation, extinction and trait transition rates across diverse phylogenies of specialists and generalists. *Journal of Evolutionary Biology*, *29*(6), 1257-1267.

Du Brul, E. L. (1977). Early hominid feeding mechanisms. *American Journal of Physical Anthropology, 47*(2), 305-320.

Futuyma, D. J., & Moreno, G. (1988). The evolution of ecological specialization. *Annual review of Ecology and Systematics*, 207-233.

Grine, F. E., Sponheimer, M., Ungar, P. S., Lee-Thorp, J., & Teaford, M. F. (2012). Dental microwear and stable isotopes inform the paleoecology of extinct hominins. *American Journal of Physical Anthropology, 148*(2), 285-317.

Hansen, T. F. (2003). Is modularity necessary for evolvability?: Remarks on the relationship between pleiotropy and evolvability. *Biosystems*, *69*(2-3), 83-94.

Hansen, T. F., & Houle, D. (2008). Measuring and comparing evolvability and constraint in multivariate characters. *Journal of Evolutionary Biology*, *21*(5), 1201-1219.

Jablonski, D. (2022). Evolvability and macroevolution: overview and synthesis. *Evolutionary Biology*, *49*(3), 265-291.

Kay, R. F. (1977). The evolution of molar occlusion in the Cercopithecidae and early catarrhines. *American Journal of Physical Anthropology, 46*(2), 327-352.

Lande, R. (1979). Quantitative genetic analysis of multivariate evolution, applied to brain: body size allometry. *Evolution*, 402-416.

Lucas, P. W. (2004). *Dental Functional Morphology: How Teeth Work*. New York: Cambridge University Press.

Lucas, P. W., Omar, R., Al-Fadhalah, K., Almusallam, A. S., Henry, A. G., Michael, S., . . . Atkins, A. G. (2013). Mechanisms and causes of wear in tooth enamel: implications for hominin diets. *Journal of the Royal Society Interface, 10*(80), 20120923. doi:10.1098/rsif.2012.0923

Meldrum, D. J., & Kay, R. F. (1997). Nuciruptor rubricae, a new pitheciin seed predator from the Miocene of Colombia. *American Journal of Physical Anthropology*, *102*(3), 407-427.

Raia, P., Carotenuto, F., Mondanaro, A., Castiglione, S., Passaro, F., Saggese, F., . . . Fortelius, M. (2016). Progress to extinction: increased specialisation causes the demise of animal clades. *Scientific Reports, 6*, 30965. doi:10.1038/srep30965

Robinson, J. T. (1954). The genera and species of the australopithecinae. *American Journal of Physical Anthropology, 12*(2), 181-200. doi:10.1002/ajpa.1330120216

Robinson, J. T. (1963). Adaptive radiation in the Australopithecines and the origin of man. In F. C. Howell & F. Bourliere (Eds.), *African Ecology and Human Evolution* (pp. 385-416). Chicago: Aldine.

Rolian, C. (2020). Ecomorphological specialization leads to loss of evolvability in primate limbs. *Evolution, 74*(4), 702-715.

Schlaepfer, M. A., Runge, M. C., & Sherman, P. W. (2002). Ecological and evolutionary traps. *Trends in Ecology & Evolution, 17*(10), 474-480.

Schluter, D. (1996). Adaptive radiation along genetic lines of least resistance. *Evolution*, *50*(5), 1766-1774.

Scott, J. E., McAbee, K. R., Eastman, M. M., & Ravosa, M. J. (2014). Experimental perspective on fallback foods and dietary adaptations in early hominins. *Biology Letters*, *10*(1), 20130789.

Scott, R. S., Ungar, P. S., Bergstrom, T. S., Brown, C. A., Grine, F. E., Teaford, M. F., & Walker, A. (2005). Dental microwear texture analysis shows within-species diet variability in fossil hominins. *Nature*, *436*(7051), 693-695.

Smith, A. L., Benazzi, S., Ledogar, J. A., Tamvada, K., Pryor Smith, L. C., Weber, G. W., . . . Strait, D. S. (2015). The feeding biomechanics and dietary ecology of *Paranthropus boisei*. *The Anatomical Record, 298*(1), 145-167. doi:doi:10.1002/ar.23073

Sponheimer, M., Daegling, D. J., Ungar, P. S., Bobe, R., & Paine, O. C. C. (2023). Problems with *Paranthropus*. *Quaternary International, 650*, 40-51. doi:10.1016/j.quaint.2022.03.024

Strait, D. S., Constantino, P., Lucas, P. W., Richmond, B. G., Spencer, M. A., Dechow, P. C., . . . Ledogar, J. A. (2013). Viewpoints: Diet and dietary adaptations in early hominins: The hard food perspective. *American Journal of Physical Anthropology, 151*(3), 339-355. doi:doi:10.1002/ajpa.22285

Taylor, A. B., & Vinyard, C. J. (2009). Jaw-muscle fiber architecture in tufted capuchins favors generating relatively large muscle forces without compromising jaw gape. *Journal of Human Evolution, 57*(6), 710-720. doi:10.1016/j.jhevol.2009.06.001

Van Casteren, A., Strait, D. S., Swain, M. V., Michael, S., Thai, L. A., Philip, S. M., . . . Shekeban, A. (2020). Hard plant tissues do not contribute meaningfully to dental microwear: Evolutionary implications. *Scientific Reports, 10*(1), 582.

Van der Merwe, N. J., Masao, F. T., & Bamford, M. K. (2008). Isotopic evidence for contrasting diets of early hominins Homo habilis and Australopithecus boisei of Tanzania. *South African Journal of Science*, *104*(3), 153-155.

Vrba, E. S. (1980). Evolution, species and fossils: how does life evolve?. *South African Journal of Science*, *61*(2), 61-84.

Vrba, E. S. (1985). Ecological and adaptive changes associated with early hominid evolution. In E. Delson (Ed.), *Ancestors: the hard evidence* (pp. 63-71). New York: A.R. Liss.

Walker, A. (1981). Dietary hypotheses and human evolution. *Philosophical Transactions of the Royal Society of London. Series B, Biological Sciences, 292*(1057), 57-64.

Wood, B., & Strait, D. (2004). Patterns of resource use in early *Homo* and *Paranthropus*. *Journal of Human Evolution, 46*(2), 119-162. doi:https://doi.org/10.1016/j.jhevol.2003.11.004

Wright, B. W. (2005). Craniodental biomechanics and dietary toughness in the genus *Cebus*. *Journal of Human Evolution, 48*(5), 473-492. doi:10.1016/j.jhevol.2005.01.006

Wynn, J. G., Alemseged, Z., Bobe, R., Grine, F. E., Negash, E. W., & Sponheimer, M. (2020). Isotopic evidence for the timing of the dietary shift toward C_4 foods in eastern African *Paranthropus*. *Proceedings of the National Academy of Sciences, 117*(36), 21978-21984. doi:10.1073/pnas.2006221117

Ungar, P. (1998). Dental allometry, morphology, and wear as evidence for diet in fossil primates. *Evolutionary Anthropology*, *6*(6), 205-217.

Ungar, P. S. (2007). Dental functional morphology. *Evolution of the human diet: the known, the unknown, and the unknowable. Oxford University Press, Oxford*, 39-55.

Ungar, P. S., Grine, F. E., & Teaford, M. F. (2008). Dental microwear and diet of the Plio-Pleistocene hominin *Paranthropus boisei*. *PLoS One, 3*(4), e2044. doi:10.1371/journal.pone.0002044

Ungar, P. S., & Lucas, P. W. (2010). Tooth form and function in biological anthropology. *A companion to biological anthropology*, 516-529.

Ungar, P. S., & Hlusko, L. J. (2016). The evolutionary path of least resistance. *Science, 353*(6294), 29-30. doi:10.1126/science.aaf8398

Xia, J., Zheng, J., Huang, D., Tian, Z. R., Chen, L., Zhou, Z., . . . Qian, L. (2015). New model to explain tooth wear with implications for microwear formation and diet reconstruction. *Proceedings of the National Academy of Sciences, 112*(34), 10669-10672. doi:10.1073/pnas.1509491112

Xu, Q., Harries, K., Li, X., Liu, Q., & Gottron, J. (2014). Mechanical properties of structural bamboo following immersion in water. *Engineering Structures, 81*, 230-239.

Ziscovici, C., Lucas, P. W., Constantino, P. J., Bromage, T. G., & Van Casteren, A. (2014). Sea otter dental enamel is highly resistant to chipping due to its microstructure. *Biology letters, 10*(10), 20140484.
